# Autophagy Activation Clears ELAVL1/HuR-Mediated Accumulation of SQSTM1/p62 during Proteasomal Inhibition in Human Retinal Pigment Epithelial Cells

**DOI:** 10.1371/journal.pone.0069563

**Published:** 2013-07-29

**Authors:** Johanna Viiri, Marialaura Amadio, Nicoletta Marchesi, Juha M. T. Hyttinen, Niko Kivinen, Reijo Sironen, Kirsi Rilla, Saeed Akhtar, Alessandro Provenzani, Vito Giuseppe D'Agostino, Stefano Govoni, Alessia Pascale, Hansjurgen Agostini, Goran Petrovski, Antero Salminen, Kai Kaarniranta

**Affiliations:** 1 Department of Ophthalmology, Institute of Clinical Medicine, University of Eastern Finland, Kuopio, Finland; 2 Department of Drug Sciences, Pharmacology Section, University of Pavia, Pavia, Italy; 3 Institute of Clinical Medicine, Pathology and Forensic Medicine, University of Eastern Finland, Kuopio, Finland; 4 Department of Clinical Pathology, Kuopio University Hospital, Kuopio, Finland; 5 Biocenter Kuopio and Cancer Center of Eastern Finland, University of Eastern Finland, Kuopio, Finland; 6 Department of Anatomy, School of Medicine, Institute of Biomedicine, University of Eastern Finland, Kuopio, Finland; 7 Department of Optometry and Vision Sciences College of Applied Medical Sciences, King Saud University, Riyadh, Saudi Arabia; 8 Laboratory of Genomic Screening, Centre for Integrative Biology, University of Trento, Trento, Italy; 9 Department of Ophthalmology, University Eye Hospital, Albert-Ludwigs University of Freiburg, Freiburg im Breisgau, Germany; 10 Department of Ophthalmology, Medical and Health Science Center, University of Debrecen, Debrecen, Hungary; 11 Stem Cells and Eye Research Laboratory, Department of Biochemistry and Molecular Biology, Medical and Health Science Center, University of Debrecen, Debrecen, Hungary; 12 Department of Neurology, Institute of Clinical Medicine, University of Eastern Finland, Kuopio, Finland; 13 Department of Neurology, Kuopio University Hospital, Kuopio, Finland; 14 Department of Ophthalmology, Kuopio University Hospital, Kuopio, Finland; University of Florida, United States of America

## Abstract

Age-related macular degeneration (AMD) is the most common reason of visual impairment in the elderly in the Western countries. The degeneration of retinal pigment epithelial cells (RPE) causes secondarily adverse effects on neural retina leading to visual loss. The aging characteristics of the RPE involve lysosomal accumulation of lipofuscin and extracellular protein aggregates called “drusen”. Molecular mechanisms behind protein aggregations are weakly understood. There is intriguing evidence suggesting that protein SQSTM1/p62, together with autophagy, has a role in the pathology of different degenerative diseases. It appears that SQSTM1/p62 is a connecting link between autophagy and proteasome mediated proteolysis, and expressed strongly under the exposure to various oxidative stimuli and proteasomal inhibition. ELAVL1/HuR protein is a post-transcriptional factor, which acts mainly as a positive regulator of gene expression by binding to specific mRNAs whose corresponding proteins are fundamental for key cellular functions. We here show that, under proteasomal inhibitor MG-132, ELAVL1/HuR is up-regulated at both mRNA and protein levels, and that this protein binds and post-transcriptionally regulates *SQSTM1/p62* mRNA in ARPE-19 cell line. Furthermore, we observed that proteasomal inhibition caused accumulation of SQSTM1/p62 bound irreversibly to perinuclear protein aggregates. The addition of the AMPK activator AICAR was pro-survival and promoted cleansing by autophagy of the former complex, but not of the ELAVL1/HuR accumulation, indeed suggesting that SQSTM1/p62 is decreased through autophagy-mediated degradation, while ELAVL1/HuR through the proteasomal pathway. Interestingly, when compared to human controls, AMD donor samples show strong SQSTM1/p62 rather than ELAVL1/HuR accumulation in the drusen rich macular area suggesting impaired autophagy in the pathology of AMD.

## Introduction

Age-related macular degeneration (AMD) is the most common eye disease leading to visual impairment in the elderly in the developed countries [1]. The disease affects the central retina called the macula, the area that is responsible for the most important sharp and colour vision [2]. AMD is associated with aging, hereditary background, smoking, hypertension, hypercholesterolemia, arteriosclerosis, obesity and unhealthy diet. In global terms, 50 million people are affected by AMD with one third of them suffering severe visual loss [3], [4]. It is estimated that the number of AMD patients will triple during the next decades due to increased numbers of aged people [5].

Primarily AMD is characterized by degeneration of the macular retinal pigment epithelial (RPE) cells that secondarily leads to cell death of photoreceptors (rods and cones) and visual loss [6]. AMD has a progressive character and may develop into either a dry (non-exudative) or wet (exudative) form [7], [8]. Neovascularization, sprouting from the choriocapillaris into the retina, is one of the clinical hallmarks of wet AMD. The dry form of the disease is more prevalent and it accounts for as many as 90% of all cases. At present, no effective cure is available for dry AMD, although anti-oxidants and omega-fatty acids have been shown to have preventive properties in certain AMD patient groups [9], [10]. In wet AMD, monthly applications of intravitreal injections of anti-VEGF antibodies have been used to suppress the activity of neovascularization [11].

AMD pathogenesis involves chronic oxidative stress, increased accumulation of lipofuscin in the lysosomes of RPE cells, as well as extracellular drusen formation and presence of chronic inflammation [2], [12], [13]. The ability to prevent the accumulation of cytotoxic protein aggregates via autophagy may be decreased in aged post mitotic RPE cells leading to degenerative changes.

Autophagy is basic catabolic mechanism which ”self eats” cellular components that are unnecessary or dysfunctional to the cell [14]. Autophagy comprises three intracellular pathways in eukaryotic cells, which are macroautophagy (hereafter referred to as autophagy), microautophagy and chaperone-mediated autophagy [15]. Apart from its important role in cellular homeostasis, autophagy is also triggered as an adaptive response during AMD-associated stress conditions [2], [6], [14]–[17]. Autophagy process begins with the formation of isolation membranes called phagophores; these latter then become elongated and surround portions of cytoplasm containing oligomeric protein complexes and organelles to form mature double membrane autophagosomes. The autophagosomes fuse with the lysosomes and their content is then degraded by lysosomal enzymes. Failure of autophagy in aged postmitotic cells, including RPE cells, can result in accumulation of aggregate-prone proteins, cellular degeneration and finally cell death [6].

SQSTM1/p62 (Sequestosome 1) is the best-characterized and ubiquitously expressed autophagy receptor that connects proteasomal clearance with lysosomes [18]–[22]. Alleviation of autophagy is usually accompanied by an accumulation of SQSTM1/p62 mostly in large perinuclear aggregates or inclusion bodies which are also positive for ubiquitin, as reported in numerous neurodegenerative diseases (such as Alzheimer’s disease, Parkinson’s disease, and Huntington disease) [23]–[28].

The evidence indicates that *SQSTM1/p62* is a stress response gene strongly induced at mRNA and protein levels by the exposure to various oxidative stimuli and proteasomal inhibitors [29]–[32].

One of the most important post-transcriptional mechanisms involves ELAVL1/HuR (embryonic lethal, abnormal vision, Drosophila)-like 1 (Hu antigen R) protein, acting mainly as a positive regulator of gene expression by binding to and increasing the stability and/or translation of specific mRNAs whose corresponding proteins are fundamental for key cellular functions. It is noteworthy that ELAVL1/HuR, and in general ELAV family, participates in various physio-pathological processes where oxidation and stress play a primary role [33]–[35]. One of the main aims of our study was to investigate whether *SQSTM1/p62* mRNA would be a target of ELAVL1/HuR protein, and whether the binding between ELAVL1/HuR protein and *SQSTM1/p62*transcript exists in human RPE cells. We also investigated how proteasomal and autophagy modulation affects *SQSTM1/p62* and *ELAVL1/HuR* expression. Moreover, their presence in drusen rich human cadaver AMD samples was studied.

## Materials and Methods

### Ethics Statement

The study was approved by the Ethics Committee of the Freiburg University Hospital, the University of Debrecen and the tenets of the Declaration of Helsinki were followed for human material. Participants provided their written informed consent for the human material in this study.

### Cell Culture and Treatments

ARPE-19 human RPE cells were obtained from the American Type Culture Collection (ATCC). The cells were grown to confluence in a humidified 10% CO_2_ atmosphere at 37°C in Dulbecco’s MEM/Nut MIX F-12 (1∶1) medium (Life Technologies, 21331) containing 10% inactivated fetal bovine serum (Hyclone, SV30160-03), 100 units/ml penicillin and 100 µg/ml streptomycin (Lonza, DE17-602E) and 2 mM L-glutamine (Lonza, BE17-605E). In proteasome experiments, the cells were exposed to 5 µM MG-132 proteasome inhibitor (Calbiochem, 474790) for 0,5 hours, 3 hours, 12 hours and 24 hours (h). The cells were exposed to 50 nM bafilomycin A1 (Sigma, B1793) for 24 h. Additionally, the cells were treated with the AMPK (AMP-activated protein kinase) activator, AICAR 2 mM (5-aminoimidazole-4-carboxyamide ribonucleoside, Toronto Research Chemical, A611700) for 0,5 h, 3 h, 12 h and 24 h. Furthermore, the autophagy was induced with starvation by culturing cells in fetal bovine serum free medium for 0,5 h, 3 h, 12 h and 24 h.

### Cellular Fractionation and Western Blotting

The nuclear extract kit (40010) from Active motif was used for the preparation of nuclear and cytoplasmic extracts from exposed ARPE-19 cells. Proteins were collected according to the manufacturer’s protocol. The whole cell extracts were prepared in M-PER® (Mammalian Protein Extraction Reagent, Thermo Scientific, 78501) according to the manufacturer’s protocol.

Proteins were diluted in 2X sodium dodecyl sulphate (SDS) protein gel loading solution, boiled for 5 minutes, separated on 12% SDS-polyacrylamide gel electrophoresis (SDS-PAGE) and processed following standard procedures. The mouse monoclonal antibodies were diluted as follows: the anti-ELAVL1/HuR (anti-ELAVL/Hu) antibody (Santa Cruz Biotech, sc-5261) at 1∶1000; the anti-SQSTM1/p62 (anti-p62) antibody (Santa Cruz Biotech, sc-28359) at 1∶1000; the anti-MAP1LC3A/LC3 (anti-LC3, microtubule-associated protein light chain 3A) antibody (Cell Signaling, 3868) at 1∶1000 and the anti-α-tubulin (Sigma, T9026) at 1∶1000. The nitrocellulose membranes signals were detected by chemiluminescence. Experiments were performed in duplicate for each different cell preparation and α-tubulin was used to normalize the data. Statistical analysis of western blot data was performed on the densitometric values obtained with the NIH image software 1.61 (downloadable at http://rsb.info.nih.gov/nih-image) and Quantity One® software.

### Fusion Plasmid Constructs

Human MAP1LC3A (LC3, light chain 3, NCBI gene bank no. AF303888) was amplified from DNase-treated (DNase I, Roche, 04716728001) total RNA extracted (Eurozol reagent, Euroclone, EMR055100) from human ARPE-19 cells. Initially, mRNA was reverse-transcribed (MultiScribe reverse transcriptase, Applied Biosystems, 4311235), and the MAP1LC3A/LC3 open reading frame (ORF) was amplified with a high-fidelity DNA polymerase (Phusion Hot start DNA polymerase, Finnzymes, F-530S). The following primers were used: sense 5′-ATA *CTCGAG* at **ATG** CCG TCG GAG AAG A and reverse 5′-TGT *AAG CTT* g **TTA** CAC TGA CAA TTT CAT CCC. The restriction sites for XhoI and HindIII are in italics. The translation initiation and termination sites are in boldface. The additional bases enabling in-frame cloning are in minuscules. The sticky ends for the amplified MAP1LC3A/LC3 ORFs as well as for the multiple cloning site of the vector pDendra2-C (Evrogen, FP821) were produced with restriction endonucleases XhoI and Hind III (MBI Fermentas, ER0691 and ER0501. respectively) [36]. Ligated (T4 DNA Ligase, Roche, 10481220001) DNA, forming a fusion gene of Dendra2 and human MAP1LC3A/LC3 was transfected into competent DH5α *E. coli* cells, which were prepared using the protocol of Inoue and others and then cultured and purified [37], [38].The integrity of the construct, named hereafter as pDendra2-hLC3 (MAP1LC3A/LC3), was determined initially by restriction endonuclease digestion analysis and finally sequencing of both the junction sites and the entire inserted MAP1LC3A/LC3 ORF.

Human Sequestosome 1, SQSTM1 (p62), NCBI gene bank no. NM_003900) was similarly inserted into the pDendra2 vector. The ORF insert was initially purchased from RZPD (Deutsches Ressourcenzentrum für Genomforschung, IRAUp969A0698D). The insert was amplified with the following primers: sense 5′-ATA *CTC GAG* at **ATG** GCG TCG CTC ACC; reverse 5′-TAT *AAG CTT* a **TCA** CAA CGG CGG GGG ATG. The restriction sites for XhoI and HindIII are shown in italics and the translation initiation and termination sites are in boldface. The additional bases enabling in-frame cloning are in minuscules. The final plasmid was named as pDendra2-hp62 (SQSTM1/p62).

### Transfections and Confocal Imaging of pDendra2-hLC3 (MAP1LC3A/LC3) and pDendra2-hp62 (SQSTM1/p62)

ARPE-19 cells were cultured in 8-well plates (μ-Slide, ibiTreat, tissue culture treated, Ibidi, 80826) in a volume of 200 µl to a subconfluent density. In all transfection experiments, ExGen 500 in vitro Transfection Reagent (MBI Fermentas, R0511) was used and its protocol followed. The DNA content was 500 ng per single well and the transfection treatment lasted for 24 h. Chemical treatments with 5 µM MG-132 and/or 2 mM AICAR followed for 24 h in fresh medium.

Before the microscopic evaluation, the medium was always changed to a fresh medium without treatment. The nuclei were stained with DRAG5™ (Biostatus, DR50050), diluted to 1/1000 in phosphate-buffered saline. The fluorescent images were obtained with a Zeiss Axio Observer inverted microscope (63×NA 1,4 oil objective) equipped with Zeiss LSM 700 confocal module (Carl Zeiss Microimaging). The change in the Dendra2 color from green to red was achieved by treatment of the cells with 405 nm UV-light [37].In the live cell imaging, a Zeiss XL-LSM S1 incubator with temperature and CO_2_ control was utilized. ZEN 2009 software (Carl Zeiss) was used for image processing.

### Attenuation of the *ELAVL1/HuR* gene Expression by RNA Interference

A control siRNA (NEG-siRNA) having no known homology with any gene (Ambion, AM4641) and siRNA designed for the human *ELAVL1/HuR* gene (Ambion, s4610) were used. The *ELAVL1/HuR* siRNA has sense sequence GCG UUU AUC CGG UUU GAC ATT, and the binding site is at the bases 615–633 of the human *ELAVL1/HuR* gene (NCBI gene bank no. NM_001419) in exon 2, near to the 5′-end of the coding region. siRNAs were transfected into ARPE-19 cells using siPORT™Amine transfection reagent (Ambion, AM4502) following the manufacturer’s instructions. Cells were grown as described above. The concentration of siRNAs in cell cultures was 30 nM and duration of siRNA treatment was held for 24 h, followed by MG-132 treatment (5 µM) for 24 h. The decrease of *ELAVL1/HuR* expression was monitored using real time quantitative RT-PCR and western blotting.

### Immunoprecipitation Followed by RNA Extraction

Immunoprecipitation on RPE cytoplasmic fractions was performed according to a previously published protocol with minor modifications [39]. Briefly, immunoprecipitation was performed at room temperature for 2 h using 1 µg of anti-Hu antibody per 50 µg of proteins diluted with an immunoprecipitation buffer (50 mM Tris pH 7.4, 150 mM NaCl, 1 mM MgCl_2_, 0.05% Igepal, 20 mM EDTA, 100 mM DTT, protease inhibitor cocktail and an RNAase inhibitor) in the presence of 50 µl of protein A/G plus agarose (Santa Cruz Biotech, sc-2003). The samples were finally subjected to RNA extraction. The negative control was obtained under the same conditions, but in the presence of an irrelevant antibody with the same isotype of the specific immunoprecipitating antibody. One hundred microliters of the immunoprecipitation mix were immediately collected from each sample and used as “input signals” to normalize the RT-PCR data.

### Real-time Quantitative RT-PCR

RNA was extracted from total homogenates, immunoprecipitated pellets and relative “input signals” by using RNeasy Micro Plus Kit (Qiagen, 74034). The reverse transcription was performed following standard procedures. PCR amplifications were carried out using the Lightcycler instrument (Roche Molecular Biochemicals) in the presence of QuantiTect SYBR Green PCR mix (Qiagen, 204143) with primers designed by using the PRIMER3 software (www-genome.wi.mit.edu/cgi-bin/primer/primer3_www.cg). Primer sequences were as follows: *ELAVL1/HuR*, 5′-GAGGCTCCAGTCAAAAACCA -3′ (upstream), 5′-GTTGGCGTCTTTGATCACCT -3′ (downstrem); *SQSTM1/p62*, 5′- CTGGGACTGAGAAGGCTCAC -3′ (upstream), 5′- GCAGCTGATGGTTTGGAAAT -3′ (downstream); RPL6, 5′- AGATTACGGAGCAGCGCAAGATTG-3′ (upstream), 5′- GCAAACACAGATCGCAGGTAGCCC-3′ (downstream). The RPL6 mRNA was chosen as the reference on which the ELAVL1/HuR and SQSTM1/p62 values were normalized because it remained relatively stable during all the treatments and it does not bear adenine/uracil-rich elements (ARE) sequences (not shown).

In the statistical analysis the GraphPad Instat statistical package (version 3.05 GraphPad software, San Diego) was used. The data were subjected to analysis of variance (ANOVA) followed, when significant, by an appropriate test, in function of the number of samples. Differences were considered statistically significant when p values <0.05.

### Preparation of the Vector Expressing HuR cMyc-His-tagged Protein

*ELAVL1/HuR* cDNA (NM 001419 ) was obtained and amplified from MCF7-cells retro-transcribed RNA and inserted into the pCMV6-AC-Myc-His PrecisionShuttle vector (PS100006; Origene Technologies) by using the forward (5′- GCC *GCGATCGC* CATGTCTAATGGTTATGA-3′) and reverse (5′-CGT *ACGCGT* TTTGTGGGACTTGTTGG-3′) primers containing the *SgfI* and the *MluI* restriction sites (in italics), respectively. The full-length open reading frame, with the Myc-His tag-encoding sequence located at the 3′-end, was confirmed by sequencing.

Recombinant vector pCMV6-HuR (ELAVL1/HuR) was transfected (3 mg per 10 cm tissue culture dish) in HEK293T cells by using Lipofectamine® 2000 transfection reagent (Life Technologies, 11668019) according to the manufacturer’s protocol. Cells were harvested 48 h after transfection and sonicated (3×15 seconds, paused by 1 minute) with 75 of amplitude at 4°C, in binding buffer (20 mM NaH_2_PO_4_, 0.5 M NaCl, 20 mM imidazole, pH 7.4) supplemented with Protease Inhibitor Cocktail (Sigma, P8340). Recombinant ELAVL1/HuR HuR-Myc-His proteins were purified by one-step affinity chromatography on HisTrap HP columns (GE Healthcare, 17-5247-01) following the recommended protocol and were eluted with 20 mM NaH_2_PO_4_, 0.5 M NaCl, 50 mM glycine, 300 mM imidazole, 10% glycerol, pH 7.5.

### Amplified Luminescent Proximity Homogenous Assay

Amplified Luminescent Proximity Homogenous Assay (ALPHA) was applied to study the interaction between recombinant *ELAVL1/HuR* and two specific ARE-bearing RNA oligos designed from 3′UTRs of *TNFalpha* and *SQSTM1/p62*. Biotinylated single-stranded TNFalpha [40] (5′-AUUAUUUAUUAUUUAUUUAUUAUUUA-3′), SQSTM1/p62 (5′-GUUUUAAAUGACUCAUAGGUCCCUGACAUUUAGUUGAUU-3′), TNFneg (5′-ACCACCCACCACCCACCCACCACCCA-3′), SQSTM1/p62neg (5′-GCCCCAAACGACCCACAGGCCCCCGACACCCAGCCGACC-3′) RNAs were purchased from Eurofins MWG Operon and recombinant ELAVL1/HuR HuR-MYC/DDK protein (TP301562) was purified from HEK293T cells transiently transfected with pCMV6- ELAVL1/HuR. The assays were performed in 384-well white OptiPlates (PerkinElmer, 6007299) using a final volume of 25 µl and optimized by titrating both interacting partners (in order to determine the optimal protein: RNA ratio). Values out of the “hooking zone”, where quenching of the signal was due to an excess of the binding partner, were used to determine the optimal concentrations of RNA-oligos and proteins. All reagents were tested in the nanomolar range using the Alpha Screen c-Myc detection kit (Perkin Elmer 6760611C) and reacted in a buffer containing 25 mM HEPES (pH 7.4), 100 mM NaCl, and 0.1% bovine serum albumin (BSA). Briefly, recombinant ELAVL1/HuR protein (range 0.1–300 nM tested, data not shown) was incubated with a biotinylated single-stranded RNA (range 0.1–300 nM) and with anti-c-Myc Acceptor beads (20 µg/ml final concentration) and, subsequently, the reaction was placed in the dark at room temperature for 30 min. The incubation in the same conditions was extended to 90 min after addition of streptavidin Donor beads (20 µg/ml final concentration). Specific interactions were quantified on a PerkinElmer Enspire plate reader by subtracting the signal of the background (non-specific binding). Dissociation constants (K_d_) for *TNFalpha* (0.087±0.021), and SQSTM1/p62 (0.207±0.024) were determined from nonlinear regression fits of the data according to a 1-site binding model in GraphPad Prism®, version 5.0 (GraphPad Software, Inc.).

### Transmission Electron Microscopy

For transmission electron microscopy, the cells were treated with 5 µM MG-132 and/or 2 mM AICAR for 3 h, 12 h and 24 h. Cell culture samples were prefixed with 2.5% glutaraldehyde in 0.1 M phosphate buffer pH 7.4 for 2 h at RT. After 15 min washing in 0.1 M phosphate buffer, the samples were post-fixed in 1% osmium tetraoxide and 0.1 M phosphate buffer for 1 hour, and again washed with phosphate buffer for 15 min prior to standard ethanol dehydration. Subsequently, the samples were infiltrated and embedded in LX-112 resin. Polymerization was carried out at 37°C for 24 h and at 60°C for 48 h. The sections were examined with a JEM-2100F transmission electron microscope (Jeol) at 200 kV.

Aggregates and autophagic vesicles were manually counted. Eight cells were randomly selected from each group for counting aggregates and six cells for autophagic vesicles. Statistical analysis was performed using SPSS (v. 19; IBM, SPSS Inc). The significance of differences between control and treated groups was analyzed with Mann-Whitney U-test. p-values <0.05 were considered significant.

### Cytotoxicity Assay

Cytotoxicity to the AICAR and other treatments was monitored by measuring the amount of lactate dehydrogenase (LDH) enzyme from the culture medium samples. The Cyto-Tox 96 Non-Radioactive Cytotoxicity Assay kit (Promega, G1781) was used for detection according to the instructions of the manufacturer. Absorbance values after the colorimetric reaction were measured at a wavelength of 490 nm with a reference wavelength of 655 nm using a BIO-RAD Model 550 microplate reader. Statistical analysis was performed using SPSS program (v. 19; IBM SPSS Inc). The significance of differences between control and treated groups was analyzed with Mann-Whitney U-test (n = 6). p-values <0.05 were considered significant.

### Assay for Cell Death Analysis

Cell death was assessed by the Annexin-V-fluorescein isothio-cyanate. We used the Apoptosis Detection Kit (MBL, BV-K 101-4) according to the manufacturer’s recommendations; the proportion of stained Annexin-V^+^ and Annexin-V^+^/Propidium iodide^+^ cells was determined by fluorescence activated cell sorter (FACS) analysis on FACS Calibur flow cytometer (BD Biosciences Immunocytometry Systems, San Jose, CA, USA) and data were analyzed using WinMDI freeware (Joseph Trotter, La Jolla).

### Immunohistochemistry

Patients with dry AMD rich with drusen had been diagnosed based on biomicroscopy and fundus photographs in the Department of Ophthalmology of Freiburg University Hospital. The study was approved by the Ethics Committee of the Freiburg University Hospital and the tenets of the Declaration of Helsinki were followed. Eyes from two age-matched patients without clinically diagnosed AMD were used as a control; one eye was provided by the Department of Ophthalmology of Freiburg University Hospital and the other one by the Department of Ophthalmology of University of Debrecen. In both cases, the tenets of the Declaration of Helsinki were followed. Enucleated eyes from cadaver human samples were embedded in paraffin according to a routine protocol and horizontal sections (5 µm) of four eyes were immunostained for SQSTM1/p62, ubiquitin (Ub) and ELAVL1/HuR. The extent of immunopositivity in the retinal pigment epithelial cells was evaluated microscopically (no staining or positive staining) by selecting 5 mm long areas of the foveomacular, perimacular and peripheral regions. All the horizontal sections were, based to the histological anatomy, from the corneal level. Foveomacular, perimacular and peripheric areas were analysed as a pack from the same horizontal section. The stainings were done with Thermo Scientific's UltraVision LP Detection System AP Polymer & Fast Red Chromogen (TL-015-AF) kit according to the manufacturer’s instructions. The antibodies used were mouse monoclonal for SQSTM1/p62 (Santa Cruz, sc-28359), rabbit polyclonal for ubiquitin (Dako, Z0458) and the mouse monoclonal for ELAVL1/HuR (Santa Cruz, sc-5261). The dilutions were 1∶500, 1∶1100 and 1∶500 respectively. Human brain with Alzheimer's disease was used as a method control for the stainings (data not shown). After the staining samples were analyzed as described above, using a Zeiss AX10 Imager A2 (Göttingen) light microscope and a Jenoptik ProgRes C5 (Optical Systems) digital camera mounted on to the microscope. Photographs were taken with the same camera. The statistical analysis for the results was conducted with a Mann-Whitney test in the SPSS Statistics 17.0 program (IBM SPSS Inc).

## Results

### AICAR Treatment Strongly Counteracts the MG-132-induced Increase of SQSTM1/p62 Protein Levels in ARPE-19 Cells

**Figure 1:**
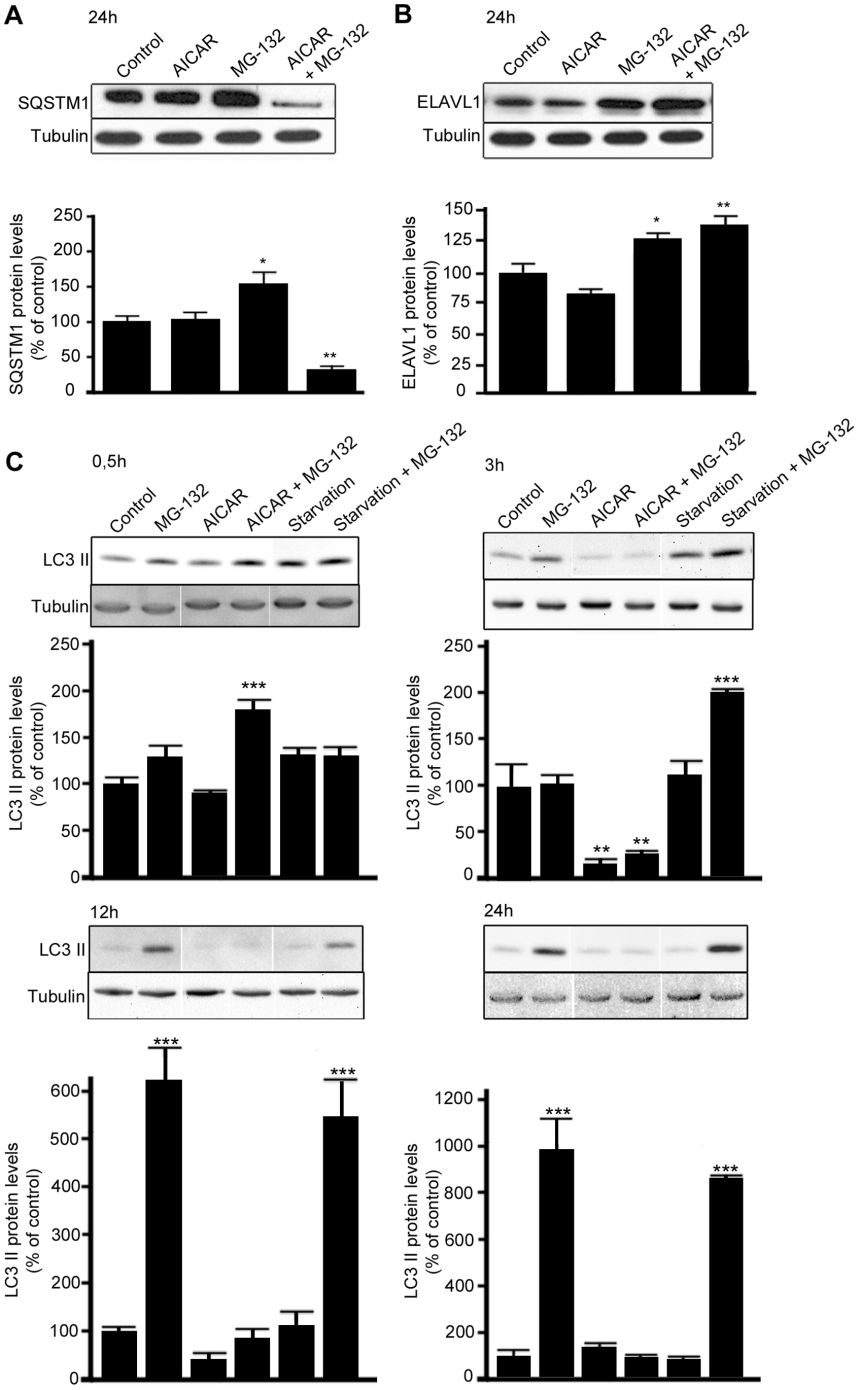
MG-132-induced increase of SQSTM1/p62 but not ELAVL1/HuR protein levels is counteracted by AICAR treatment. Representative western blotting (upper) and densitometric analysis (lower) of SQSTM1/p62 (A), ELAVL1/HuR (B) and MAP1LC3A/LC3-II (C) proteins in the total homogenates of ARPE-19 cells after starvation or/and exposure to AICAR (2 mM) or/and MG-132 (5 µM) for 0,5 h, 3 h, 12 h and 24 h. α-tubulin was used as a loading control. Control cells were exposed only to solvent (DMSO). Results are expressed as means ± S.E.M. The data were analyzed by ANOVA, followed by Dunnett’s Multiple Comparison Test; *p<0.05, **p<0.01, control vs. treated, n = 7 (SQSTM1/p62 and ELAVL1/HuR), n = 3 (MAP1LC3A/LC3-II).

In order to identify the optimal conditions to study the effects on SQSTM1/p62 protein levels, the ARPE-19 cells were first treated with the proteasome inhibitor MG-132 (5 µM) and/or the autophagy activator, AICAR (2 mM) over a time range (data not shown). Western blotting analysis revealed that a statistically significant change in total SQSTM1/p62 protein levels was detectable only after 24 h treatments, so this time point was selected for further studies. In particular, as shown in Figure 1A, the proteasome inhibitor MG-132 significantly increased total SQSTM1/p62 protein levels, while concomitant treatment with MG-132 and AICAR robustly decreased SQSTM1/p62 protein levels. In contrast, no difference in SQSTM1/p62 protein levels was detected after exposure to AICAR alone (Fig. 1A). Interestingly, by performing cellular fractionation, we found that in comparison to control cells, MG-132 evoked a significant increase in the SQSTM1/p62 protein levels mainly in the nuclear/perinuclear compartment and also in the cytoplasm (+1658%, p<0.05 and +48%, p<0.001, respectively; Fig. S1A). Analogously to SQSTM1/p62, MG-132 also significantly increased ELAVL1/HuR protein levels in the nuclear/perinuclear compartment, in the cytoplasm (+154%, p<0.001 and +64%, p<0.01 respectively; Fig. S1B) and in the total homogenate (Fig. 1B). In contrast to SQSTM1/p62, the MG-132-induced increase of ELAVL1/HuR protein was not affected by the concomitant treatment with AICAR, as reported in Figure 1B, suggesting that ELAVL1/HuR protein is not degraded via autophagy.

To confirm the capability of AICAR to induce autophagy, MAP1LC3A/LC3–II protein levels were detected after 0,5 h, 3 h, 12 h and 24 h by western blotting (Fig. 1C). Indeed, when co-treated with MG-132, AICAR clearly increased MAP1LC3A/LC3-II protein levels after 0,5 h, while after 24 h treatment MAP1LC3A/LC3 lipidation was already over (Fig. 1C), in accordance with the SQSTM1/p62 levels. We also wanted to compare AICAR response with starvation, a condition that is known as autophagy inducer. As well as for AICAR, starvation-induced autophagy required the presence of the proteasome inhibitor MG-132; conversely to AICAR, starvation-induced autophagy under proteasome inhibition begun later than AICAR (3 h vs 0,5 h) in ARPE-19 cells.

### MG-132 Treatment Triggers the Up-regulation of *SQSTM1/p62* Expression through the RNA-binding ELAVL1/HuR Protein

It has been documented that *SQSTM1/p62* is oxidative stress-related and ubiquitin-binding protein [40]. We here confirmed that 24 h MG-132 treatment induces the increase of *SQSTM1/p62* mRNA expression in the total homogenate of ARPE-19 cells (Fig. S2B). Then we wanted to determine whether a positive regulation of *SQSTM1/p62* expression occurs also at post-transcriptional level in human ARPE-19 cells, following MG-132 treatment.

**Figure 2:**
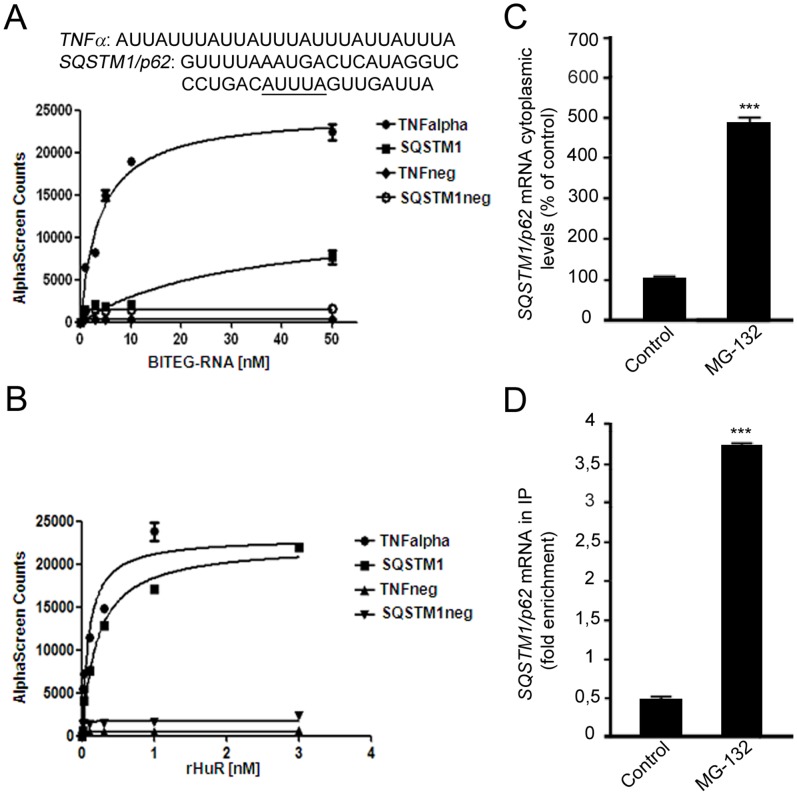
*SQSTM1/p62* transcript as a new target of the RNA-binding ELAVL1/HuR protein. (A): RNA-binding activity of ELAVL1/HuR evaluated by AlphaScreen technology. Saturation binding experiments investigated by titrating a series of biotinylated single-stranded (BITEG-) RNAs, including *TNF*neg and *SQSTM1/p62*neg, that we designated as negative controls, against 1 nM of rELAVL1/HuR. Calculated dissociation constants (K_d_) for *TNFalpha* (3.83±0.69 nM, R^2^ = 0.97), and *SQSTM1/p62* (30.85±13.22 nM R^2^ = 0.91) are indicated. The plots represent mean ± SD of two independent experiments. (B): Saturation binding experiments as function of rELAVL1/HuR concentrations against four different type of RNA-substrates at 50 nM concentration. 1 nM of rELAVL1/HuR was enough to reach saturation of the binding. The plots represent Mean ± SD of two independent experiments. (C): Effect of MG-132 exposure on *SQSTM1/p62* gene expression. Determination of *SQSTM1/p62* mRNA by real-time qPCR in human ARPE-19 cells following treatments with solvent (control) or 5 µM MG-132 for 24 hrs. *SQSTM1/p62* mRNA expression in control cells was taken as 100%. The values obtained from total cellular mRNA have been normalized to the level of RPL6 mRNA and expressed as mean ± S.E.M. ***p<0.001; Student’s t test; n = 3. (D): The binding of ELAVL1/HuR protein to *SQSTM1/p62* transcript increases in the cytoplasm following MG-132 stimulus. Fold enrichment detected by quantitative real-time RT-PCR of *SQSTM1/p62* mRNA in control and 24 h MG-132 RPE cells following immunoprecipitation with anti-ELAV antibody (IP) in the cytoplasm. ***p<0.001, Student’s t-test, n = 3. The data of SQSTM1/p62 were normalized with respect to the data obtained from immunoprecipitation with an irrelevant antibody as a negative control.

First, we analyzed the *SQSTM1/p62* sequence to identify the *cis*-acting elements and the corresponding *trans*-acting factors potentially affecting *SQSTM1/p62* mRNA stability and/or translation. A detailed primary sequence analysis of *SQSTM1/p62* revealed the presence of ARE in the 3′-untranslated region (3′-UTR), three of which (AUUUA) represent the canonical class I sequence recognized by the RNA-binding ELAV proteins. This region was consequently selected to design a probe used in protein-RNA binding AlphaScreen assay, an *in vitro* technology which can be utilized to evaluate the possible binding between ELAVL1/HuR protein and *SQSTM1/p62* mRNA. An ARE-bearing probe, a portion of TNFα 3′UTR, was employed as the positive control of the experiment since it contains well characterized ELAV-target sequences [41]. As shown in Figure 2A and 2B, ELAVL1/HuR protein physically interacts with the *SQSTM1/p62* probe, leading to the formation of a stable protein-RNA probe complex. From saturation binding experiments we calculated the dissociation constants for the two RNA probes. The resulting binding efficiency for the *SQSTM1/p62* probe was 8 times weaker than the *TNFα* probe, as it can be reasonably explained by the presence of four canonical AREs within the *TNFα* probe compared to the one present in the *SQSTM1/p62* probe.

**Figure 3:**
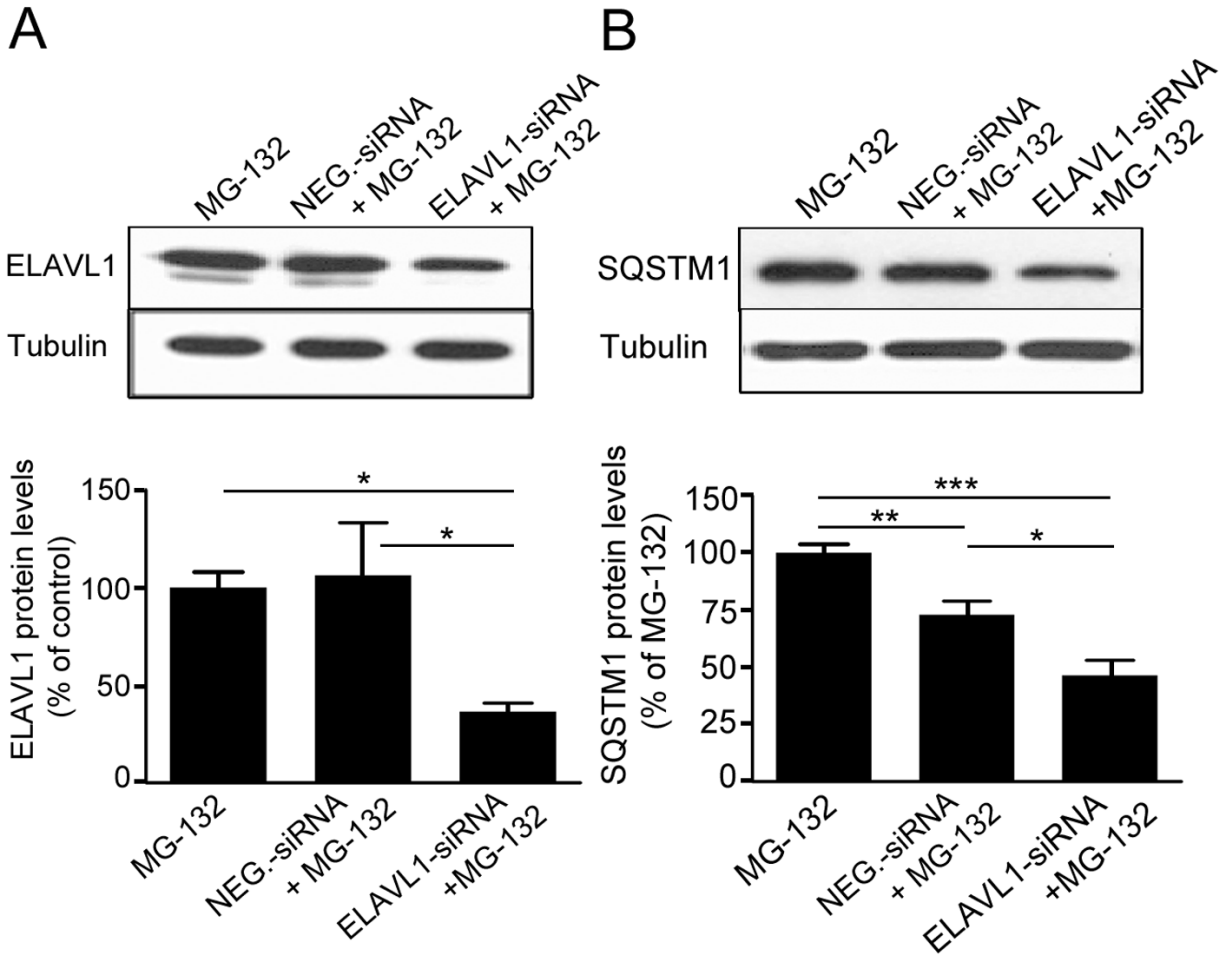
The MG-132-mediated upregulation of SQSTM1/p62 protein expression requires the specific presence of ELAVL1/HuR protein. Representative western blotting (upper) and densitometric analysis (lower) of ELAVL1/HuR (A) and SQSTM1/p62 (B) proteins in the total homogenates of ELAVL1/HuR silenced ARPE-19 cells and negative control (NEG-siRNA) cells after exposure to 5 µM MG-132 for 24 h. α-tubulin was used as a loading control. Results are expressed as means ± S.E.M. *p<0.05; **p<0.01; ***p<0.001, Tukey’s multiple comparison test; n = 5.

To confirm the finding obtained in the *in vitro* experiments and to evaluate whether the binding between ELAVL1/HuR protein and *SQSTM1/p62* mRNA exists also in our cell system, we then performed immunoprecipitation coupled with RT-real-time qPCR experiments on control and MG-132 treated cells. It was found that the MG-132-induced up-regulation of *SQSTM1/p62* mRNA in the cytoplasm of ARPE-19 cells (Fig. 2C) was accompanied by a parallel increase of the binding between ELAVL1/HuR protein and *SQSTM1/p62* mRNA in the same cellular fraction (Fig. 2D), indicating that MG-132 treatment induced a positive regulation of *SQSTM1/p62* expression that occurred also at post-transcriptional level via ELAVL1/HuR protein. To further confirm this hypothesis, we silenced *ELAVL1/HuR* expression in ARPE-19 cells by siRNA technology and then exposed these cells to MG-132 for 24 h. First, we found that MG-132 treatment strongly up-regulates *ELAVL1/HuR* mRNA expression in ARPE-19 cells (Fig. S2A). Conversely, in silenced cells, MG-132 does not affect *ELAVL1/HuR* mRNA total amount, that is indeed comparable to control levels (Fig. S2A). We then evaluated ELAVL1/HuR protein levels finding that, besides *ELAVL1/HuR* mRNA, ELAVL1/HuR-siRNA also counteracted MG-132-induced ELAVL1/HuR protein accumulation (Figure 3A). In parallel, we found that, in ELAVL1/HuR-silenced cells, the MG-132 exposure leads to an up-regulation of *SQSTM1/p62* mRNA expression in a less extent than in MG-132-treated control cells (Fig. S2B). Moreover, the MG-132-induced increase of SQSTM1/p62 protein was counteracted in ELAVL1/HuR silenced cells (Fig. 3B), for the first time suggesting that the positive regulation of *SQSTM1/p62* expression, during proteasomal inhibition, requires the specific presence of ELAVL1/HuR protein.

### AICAR Promotes the Complete Clearance of MG-132-induced Protein Aggregates, SQSTM1/MAP1LC3A (p62/LC3) Co-localization and Autophagy-related Gene Expression

**Figure 4:**
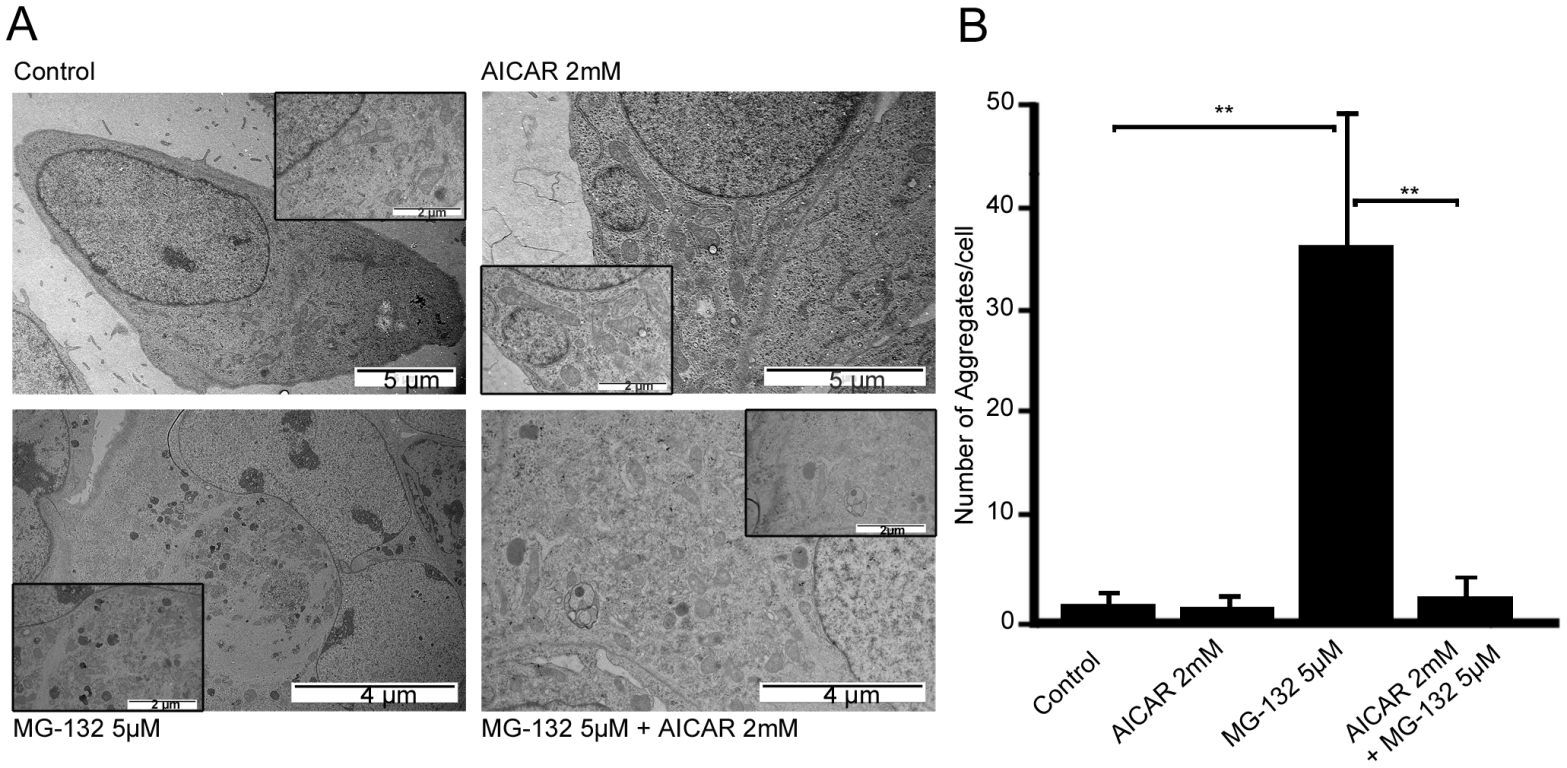
Transmission electron micrographs of ARPE-19 cells exposed to AICAR or/and MG-132 and aggregate quantification. (A) Representative transmission electron micrographs of ARPE-19 untreated control cells, cells exposed to AICAR 2 mM or/and MG-132 5 µM for 24 h. Aggregates are indicated by arrows. (B) Quantification of aggregates in ARPE-19 cells exposed to AICAR 2 mM or/and MG-132 5 µM for 24 h. Eight parallel samples were measured in all treatments. **p<0.01, Mann–Whitney.

**Figure 5:**
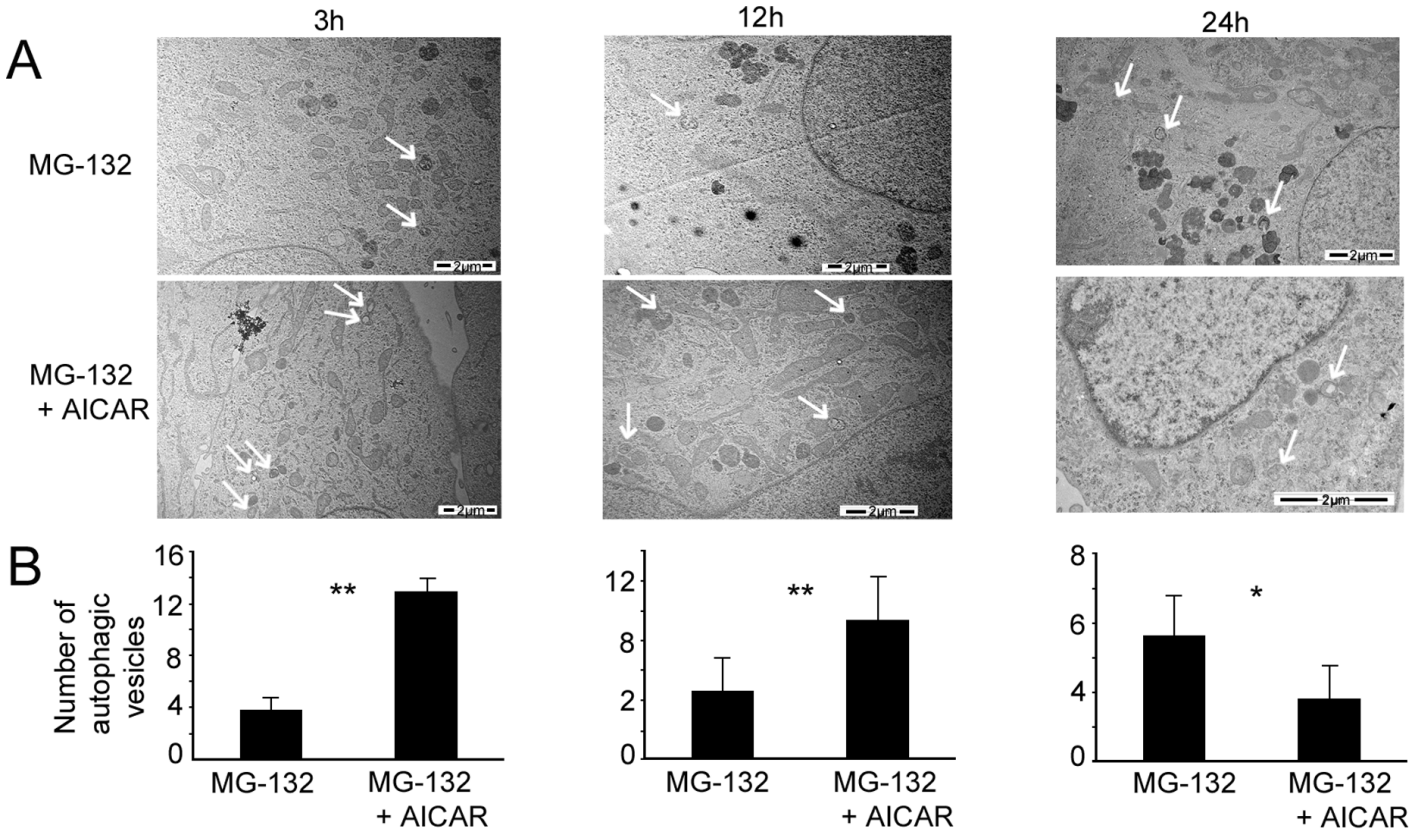
Transmission electron micrographs of ARPE-19 cells exposed to AICAR or/and MG-132 and autophagic vesicles quantification. (A) Representative transmission electron micrographs of ARPE-19 cells exposed to MG-132 5 µM solely and with AICAR 2 mM for 3 h, 12 h and 24 h. Autophagic vesicles are indicated by arrows. (B) Quantification of autophagic vesicles in ARPE-19 exposed to MG-132 5 µM solely and with AICAR 2 mM for 3 h, 12 h and 24 h. Six parallel samples were measured in all treatments. *p<0.05, **p<0.01, Mann–Whitney.

As shown in Figure 1A, SQSTM1/p62 protein levels decreased following concomitant treatment with MG-132 and AICAR, subsequently we investigated in more detail the capacity of AICAR to affect protein aggregation which normally occurs under proteasome inhibition in ARPE-19 cells. The cells were thus exposed to AICAR and MG-132 and protein aggregates were examined in a transmission electron microscope. As shown in Figure 4, AICAR was able to completely abolish the MG-132-induced protein aggregation after 24 h treatment. Moreover, there was also observed obvious reduction of aggregates after 3 h and 12 h AICAR- and MG-132-co-treated cells (data not shown). Consistently with Figure 1 results, the amount of autophagic vesicles increased during first 3 and 12 hours significantly when cells were treated with AICAR together with MG-132 (Fig. 5). These results show that this concomitant treatment strongly switches-on autophagy at early time and clearance is already over after one day.

**Figure 6:**
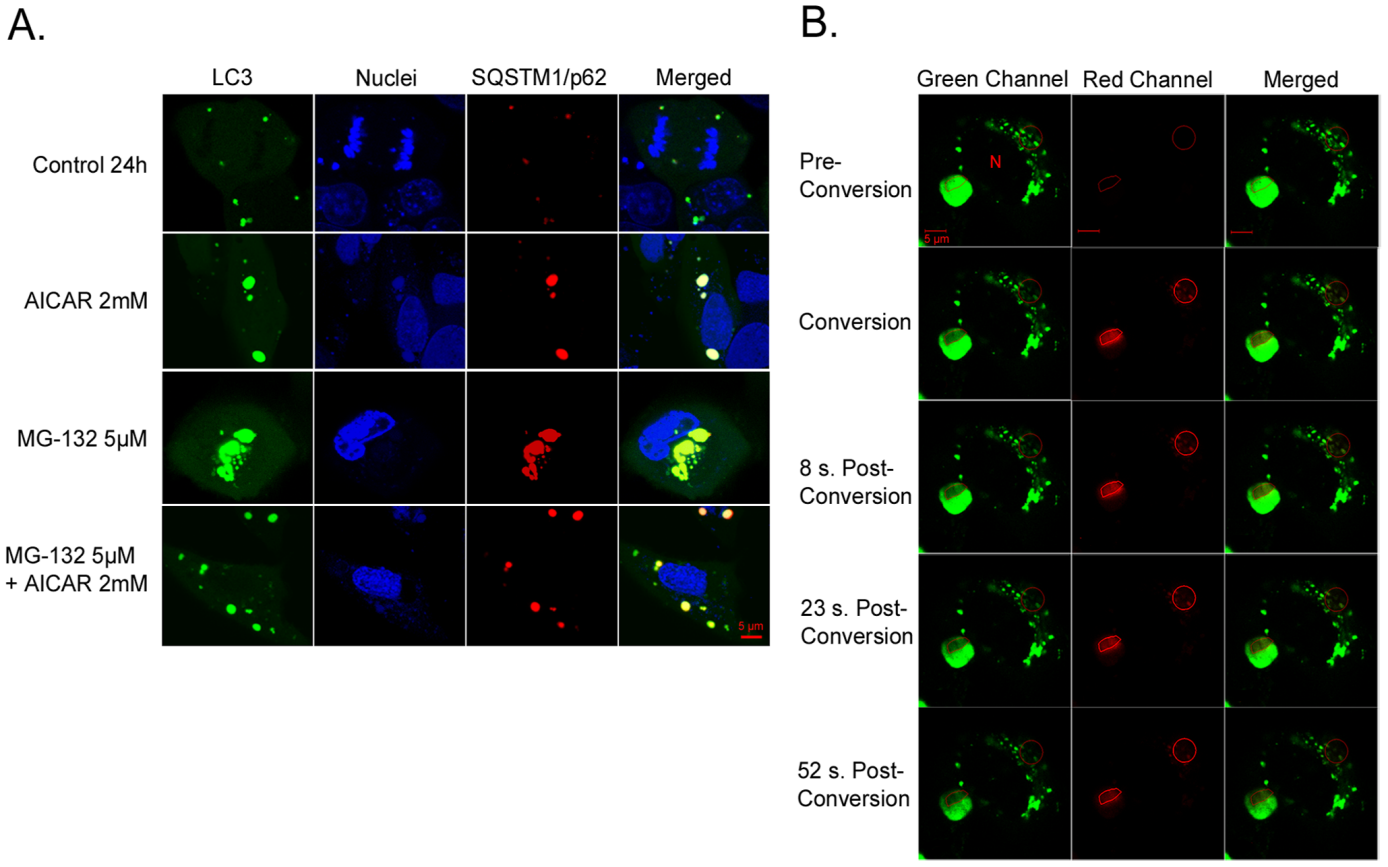
Colocalization of SQSTM1/p62 and MAP1LC3A/LC3 and position changes of SQSTM1/p62, revealed by confocal microscopy analysis. A light merge orange/yellow signal of colocalizing MAP1LC3A/LC3 (pDendra2-hLC3, green) and SQSTM1/p62 (pDsRed2-hp62, red) is detectable, usually near to the cell nuclei. Nuclei are stained with blue dye. The scale bar equals to 5 µm. (A): Confocal microscopy images of untreated control ARPE-19 cells and cells exposed to AICAR 2 mM or/and MG-132 5 µM for 24 h. A = Aggregates (B): Position of SQSTM1/p62 (pDendra2-hp62) in ARPE-19 cells, revealed by confocal microscopy analysis. SQSTM1/p62 in two different areas was photoconverted within approximately 7 sec (circled). Within 1 minute after photoconversion, the photoconverted SQSTM1/p62 is stationary. Cells have been exposed to 5 µM MG-132 for 24 h. N = Cell nucleus.

Since it is known that SQSTM1/p62 protein contains MAP1LC3A/LC3 interacting domain [42] we evaluated if SQSTM1/p62 protein would be co-localized with the autophagosome marker MAP1LC3A/LC3 under different stimuli in ARPE-19 cells. The cells were transfected with pDendra2-hLC3 (MAP1LC3A/LC3) and pDsRed2-hp62 (SQSTM1/p62) plasmids, exposed to AICAR and MG-132 as previously described in the text, and analyzed by live confocal microscopy. Figure 6A illustrates that MAP1LC3A/LC3 and SQSTM1/p62 proteins co-localized after all treatments and that a clear perinuclear accumulation of both proteins could be seen after MG-132-induced proteasome inhibition. In agreement with previous data, the cells treated concomitantly with MG-132 and AICAR exhibited a strong reduction of the perinuclear aggregates containing both MAP1LC3A/LC3 and SQSTM1/p62 proteins (Fig. 6A). In addition, we noted that MAP1LC3A/LC3 punctas were somewhat larger in AICAR-treated cells than untreated cells. This interesting observation remains still to be studied.

### Accumulated SQSTM1/p62 Protein is Stationary Following 24 hours’ Proteasome Inhibition in ARPE-19 Cells

It has been previously shown that SQSTM1/p62 protein accumulates in perinuclear aggregates that undergo autophagy clearance [18], [21]. According to our unpublished data there are proteins that have reversible binding capacity to perinuclear aggregates. Therefore, we wanted to evaluate whether binding of SQSTM1/p62 to aggregates is reversible or irreversible prior to autophagy.

In order to observe whether, under MG-132 stimulus, the increased SQSTM1/p62 protein levels were accompanied by its intracellular translocation in ARPE-19 cells, the fusion protein plasmid construct pDendra2-hp62 (SQSTM1/p62) was created and transfected to cells. Confocal microscopy was used for live cell imaging and the movement of SQSTM1/p62 protein was evaluated after photo converting pDendra2-hp62 (SQSTM1/p62) (Fig. 6B). Two areas containing SQSTM1/p62 protein were photoconverted: the intensively aggregated SQSTM1/p62 and the amorphously aggregated SQSTM1/p62. We detected no position changes of SQSTM1/p62 protein from the converted area to other parts of the cell within 1 minute, indicating that accumulated SQSTM1/p62 is stationary in ARPE-19 cells treated for 24 h.

### AICAR is Well-tolerated and Prevents Cell Death in MG-132 Long-exposed ARPE-19 Cells

Next we evaluated how well ARPE-19 cells could tolerate exposure to AICAR. Cellular toxicity was evaluated in two ways after 24 h exposure to AICAR on its own and/or with the proteasome inhibitor MG-132 by lactate dehydrogenase and Annexin-V assays. AICAR treatment, in the presence or absence of the proteasome inhibitor, did not increase lactate dehydrogenase release as compared to untreated control cells after 24 h (Fig. S3A), evidence of the good cellular tolerability towards AICAR. Annexin-V and Propidium Iodide double staining revealed that MG-132 treatment is able to induce apoptosis after prolonged (48 and 72 h) exposure; in particular, we found that proteasome inhibition clearly induced early and late apoptosis, the latter being more pronounced after 72 h of treatments (Fig. S3B). After exposure for both 48 and 72 h to MG-132, the concomitant presence of AICAR counteracted the apoptotic effects, indicating that AICAR exerted cytoprotective properties against proteasome inhibition in ARPE-19 cells, by favoring autophagy which reduced proteasome blockade-induced toxicity.

### SQSTM1/p62 Protein, but not ELAVL1/HuR Protein, is Degraded by Autophagy

**Figure 7:**
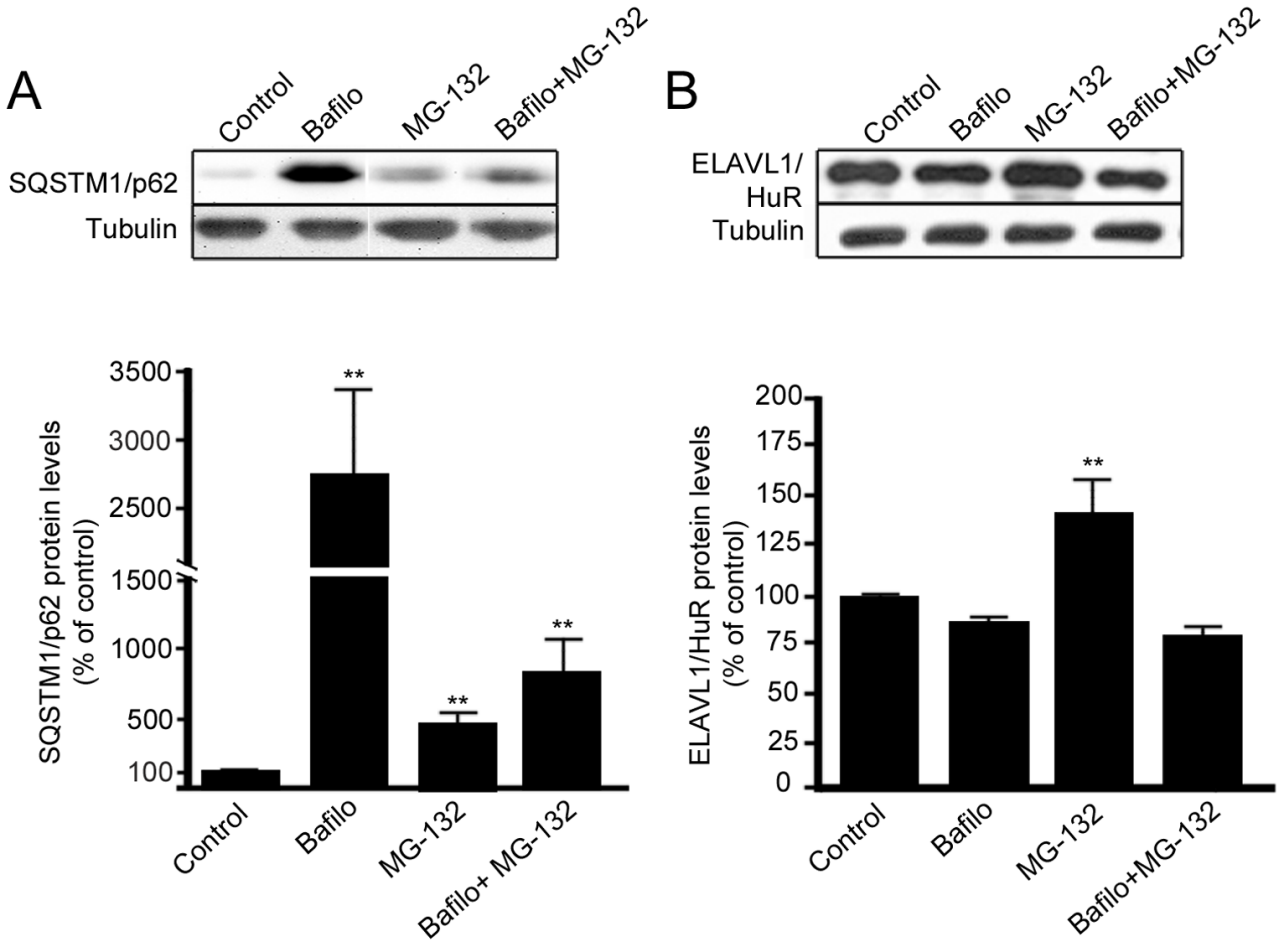
SQSTM1/p62 protein, but not ELAVL1/HuR protein, is degraded by autophagy in ARPE-19 cells. Representative western blotting and densitometric analysis of SQSTM1/p62 (A) and ELAVL1/HuR (B) proteins in the total homogenates of ARPE-19 cells after exposure to bafilomycin (50 nM) or/and MG-132 (5 µM) for 24 h. α-tubulin was used as a loading control. Results are expressed as means ± S.D. The data were analyzed by ANOVA, followed by Mann-Whitney; **p<0.01, untreated control cells vs. treated cells, n = 7.

Bafilomycin is known to be an autophagy inhibitor preventing the fusion of autophagosomes and lysosomes, although effect may change between cell types and exposure time [43]. To test how bafilomycin affects SQSTM1/p62 protein expression in ARPE-19, these cells were treated with either 50 nM bafilomycin or 5 µM MG-132 or with both agents simultaneously, and total protein extracts were analyzed by western blotting. In accordance with our previous experiments, ARPE-19 cells treated with bafilomycin displayed a dramatic increase of SQSTM1/p62 protein levels (Fig. 7A). In addition, when cells were exposed to both bafilomycin and MG-132, there was a statistically significant increase of SQSTM1/p62 protein amount compared to untreated (Fig. 7A), although to a lesser extent than with bafilomycin alone, implying that SQSTM1/p62 clearance was mediated via autophagy in ARPE-19 cells. Conversely, we did not observe any change in ELAVL1/HuR protein levels after bafilomycin (Fig. 7B), further indicating that ELAVL1/HuR protein was being degraded via proteasome.

### SQSTM1/p62 Protein Levels in the Foveo-macula Areas of AMD Patients

**Figure 8:**
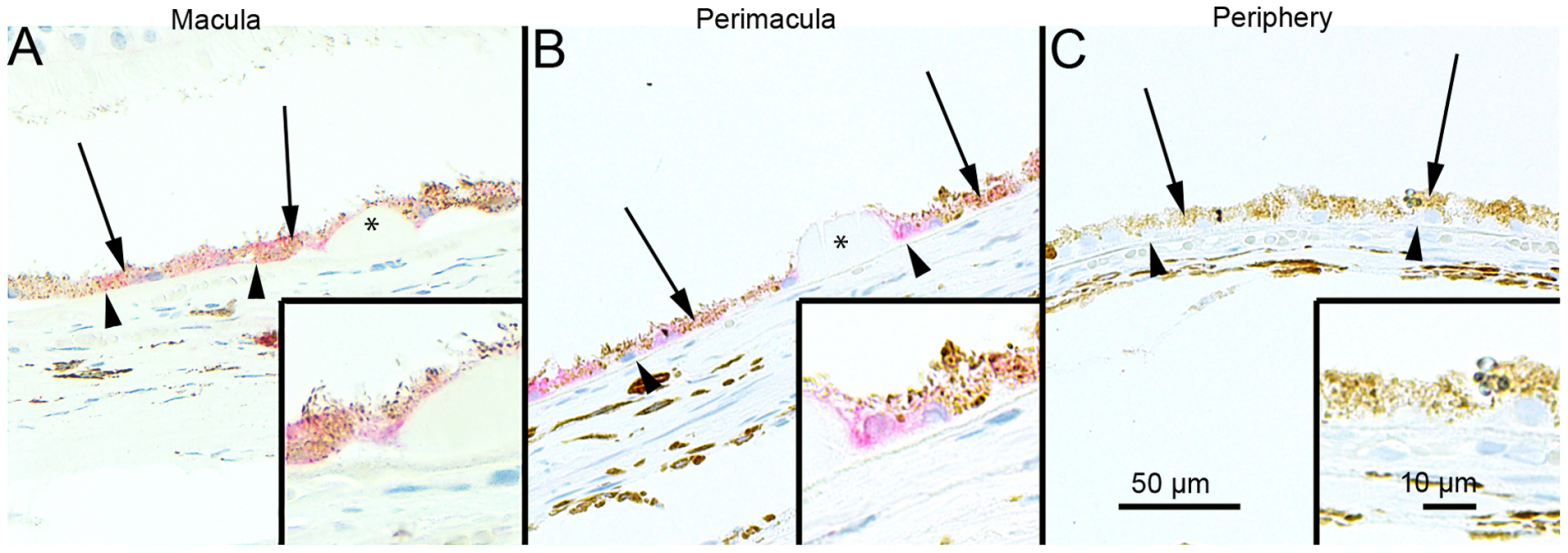
Sections of eyes with clinically diagnosed AMD immunostained for SQSTM1/p62. The extent of cytoplasmic immunopositivity in the retinal pigment epithelial cells (RPE, shown by arrows) and in the drusen was evaluated microscopically (no staining or positive staining) by selecting 5 mm long areas of foveomacular (A), perimacular (B) and peripheral (C) regions. The SQSTM1/p62 staining in the foveomacular areas was more extensive as compared to the perimacular and peripheral areas (B and C, respectively). The drusen (shown by asterisks) were mostly SQSTM1/p62 negative. The nuclei of RPE cells were SQSTM1/p62 negative. (Original magnifications of x 200 and in insets x 400; Bruch's membrane shown by arrow heads).

**Figure 9:**
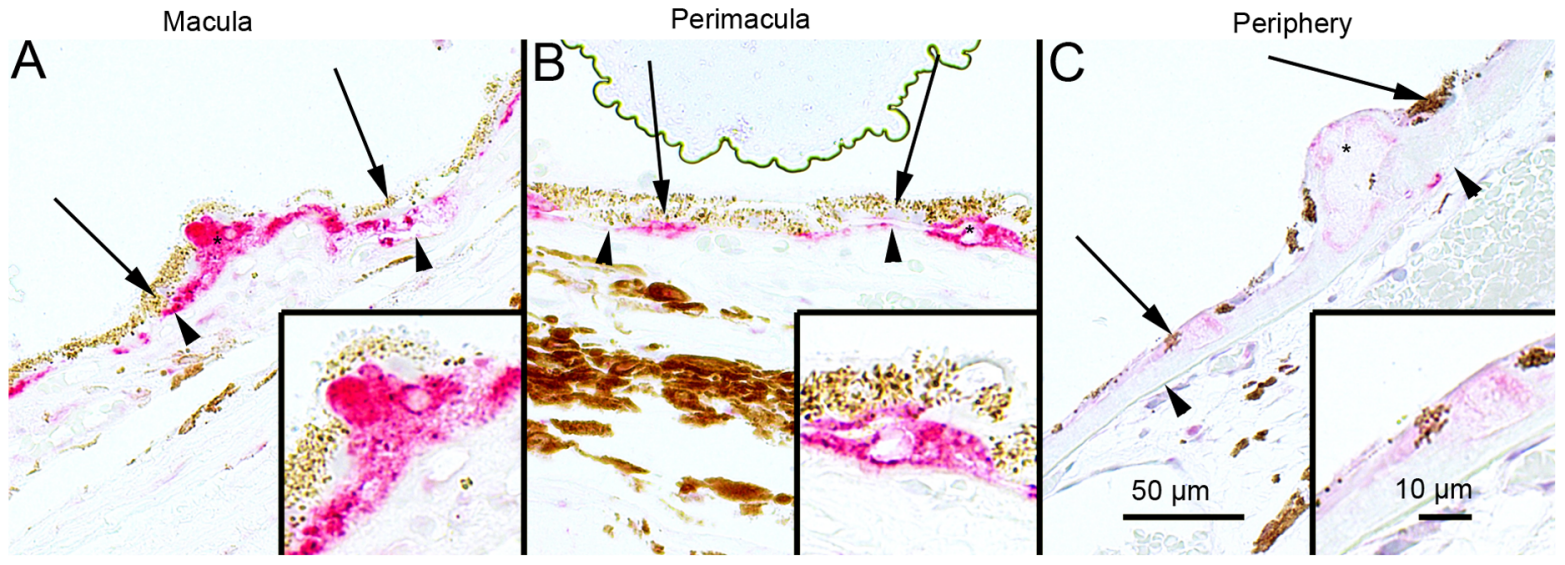
Sections of eyes with clinically diagnosed AMD immunostained for ubiquitin. The extent of Bruch’s membrane immunopositivity of the retinal pigment epithelial cells (RPE, shown by arrows) and in the drusen (asterisks) was evaluated microscopically (no staining or positive staining) by selecting 5 mm long areas of foveomacular (A), perimacular (B) and peripheral (C) regions. The uniform staining of ubiquitin in RPE cells Bruch’s membrane was observed in all these regions (arrows). There were no differences in the extent of staining between these regions in each group. The nuclei of RPE cells were mostly ubiquitin negative. Most of the drusen were strongly ubiquitin-positive (asterisks). (Original magnifications of x 200 and in insets x 400; Bruch's membrane shown by arrow heads).

**Figure 10:**
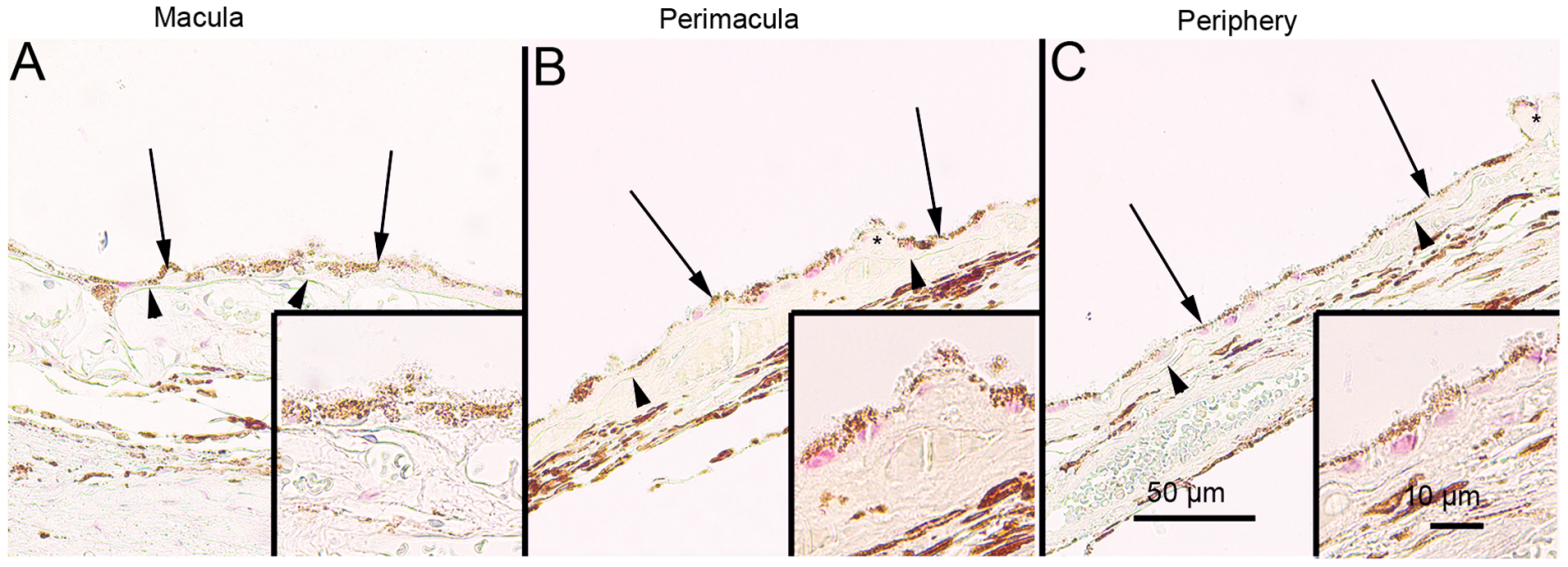
Sections of eyes with clinically diagnosed AMD immunostained for ELAVL1/HuR. The immunopositivity of the nuclei of RPE cells (shown by arrows) and the extent of immunopositivity in the drusen (asterisks) was evaluated microscopically (no staining or positive staining) by selecting 5 mm long areas of foveomacular (A), perimacular (B) and peripheral (C) regions. The foveomacular nuclei showed less extensive immunopositivity for ELAVL1/HuR than the perimacular and peripheral regions. However this difference was not statistically significant in any of the groups studied (p>0.1). The cytoplasms of RPE cells were ELAVL1/HuR negative. Most of the drusen were ELAVL1/HuR-negative. (Original magnifications of x 200 and in insets x 400; Bruch's membrane shown by arrow heads).

**Figure 11:**
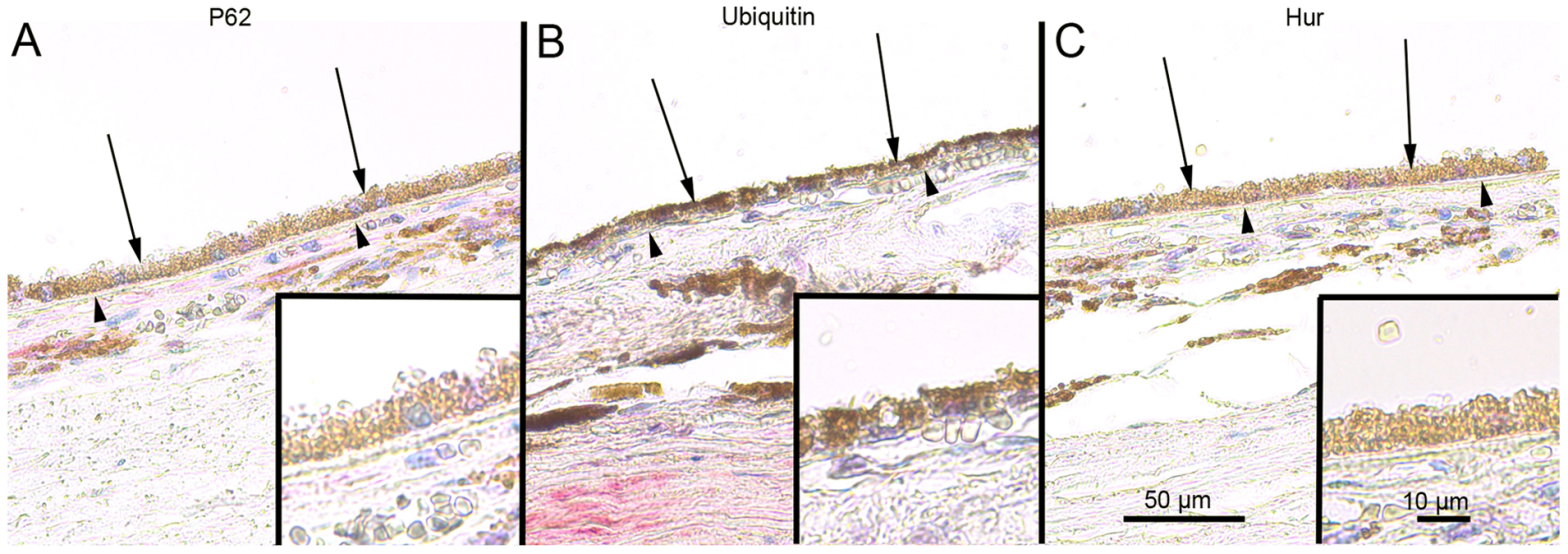
Sections of foveomacular areas of age-matched control eyes for SQSTM1/p62, ubiquitin and ELAVL1/HuR. The extent of cytoplasmic immunopositivity for SQSTM1/p62, Bruch’s membrane immunopositivity for ubiquitin and the immunopositivity of nuclei of RPE cells for ELAVL1/HuR (foveomacular areas shown; A, B and C respectively) in the RPE cells (shown by arrows) was evaluated microscopically (no staining or positive staining). For SQSTM1/p62 there were only a few immunopositive cytoplasm’s randomly distributed through-out the RPE cell layer and the nuclei were negative. Bruch’s membrane (shown by arrowheads) was immunopositive for ubiquitin occasionally through-out the RPE cell layer in very small amounts while all of the nuclei were negative. Half of the nuclei showed minor immunopositivity for ELAVL1/HuR while the cytoplasm’s of RPE cells were negative. The foveomacular areas showed no difference in immunohistochemical stainings when compared to areas of perimacular and peripheral retina in all of the proteins studied. (Original magnifications of x 200 and in insets x 400).

Since, it is uncertain that the compounds used to regulate autophagy in the cell culture model also mimic cellular aging and age-related disease we wanted to analyze these key proteins in human tissue samples. The SQSTM1/p62 staining in the foveomacular areas of the AMD patients was more extensive compared to the perimacular and peripheral areas (p<0.001), the latter representing the internal control (Fig. 8; Table 1). The drusen were mostly SQSTM1/p62 negative while the nuclei of RPE cells were SQSTM1/p62 negative. A uniform staining of ubiquitin in RPE cells and Bruch’s membrane was observed in all these regions (Fig. 9). There were no differences in the extent of ubiquitin staining between these regions in all groups (p>0.1) (Table 1). Most of the nuclei of RPE cells were ubiquitin negative and most of the drusen were strongly ubiquitin positive. The foveomacular nuclei showed less extensive immunopositivity for ELAVL1/HuR protein than the perimacular and peripheral regions (Fig. 10). However this difference was not statistically significant in any of the groups studied (p>0.1) (Table 1). The cytoplasms of RPE cells were ELAVL1/HuR negative while the drusen were mostly ELAVL1/HuR negative. We also performed the same immunohistochemistry experiments in age-matched control samples. We found that they were mostly SQSTM1/p62 negative, especially in the macular region (Fig. 11A); all of the nuclei of RPE cells were negative, and there were only a few SQSTM1/p62 immunopositive cytoplasms randomly distributed through-out the RPE cell layer. In the control patient samples Bruch’s membrane was mostly immunonegative for ubiquitin; occasionally through-out the RPE cell layer, few cytoplasms were weakly ubiquitin positive, while all of the nuclei were negative in all the regions studied (Fig. 11B). In these control sections, half of the nuclei showed weak immunopositivity for ELAVL1/HuR while the cytoplasms of RPE cells were negative through-out the RPE cell layer (Fig. 11C).

**Table 1:** Cytoplasmic SQSTM1/p62, ubiquitin and ELAVL1/HuR proteins in foveomacular, perimacular and peripheral RPE cells.

SQSTM1/p62	Macula	Perimacula	Peripheral retina
Sample	Percentage (%)	Percentage (%)	Percentage (%)
1	100	48	0
2	100	64	0
3	92	16	12
4	100	26	36
5	90	7	36
6	100	48	0
7	100	38	24
8	100	22	18
MA; SD (%)	97,8; 4,2	33,6; 19,2	15,8; 15,4
**Ubiquitin**	**Macula**	**Perimacula**	**Peripheral retina**
**Sample**	**Percentage (%)**	**Percentage (%)**	**Percentage (%)**
1	15	30	0
2	14	44	9,6
3	64	66	40
4	70	56	26
5	70	26	32
6	64	32	30
7	20	9	7,4
8	16,4	16,8	4
**MA; SD (%)**	**41,7; 27,2**	**35,0; 19,3**	**18,6; 15,1**
**ELAVL1/HuR**	**Macula**	**Perimacula**	**Peripheral retina**
Sample	Percentage (%)	Percentage (%)	Percentage (%)
1	6,7	8,7	0
2	32,6	0	66,7
3	56,4	100	61,5
4	5,6	36,4	56,2
5	30,4	50	42,5
6	38,3	35,4	27,1
7	0	0	0
8	0	0	0
MA; SD (%)	21,3; 21,1	28,8; 34,9	31,8; 28,9

P-value for SQSTM1/p62 between macular-perimacular and macular-peripheral retina groups is p<0.001. The differences among the three groups for ubiquitin and ELAVL1/HuR are not statistically significant (p>0.1, Mann-Whitney test).

Eight 5 mm long regions from macular, perimacular and peripheral retina were selected from eyes with clinically diagnosed AMD. If the optical nerve was visible, the macular area was selected next to it; if not, a region which contained most drusen was selected. Peripheral regions were selected close to the ora serrata so that it would have as few as possible drusen. The perimacular region was selected between these two so that it would contain few drusen. The extent of cytoplasmic immunopositivity was measured and its percentage in relation to the whole measurement was calculated. The statistical analysis for the results was conducted with a Mann-Whitney test. MA = mean value, SD = standard deviation.

## Discussion

**Figure 12:**
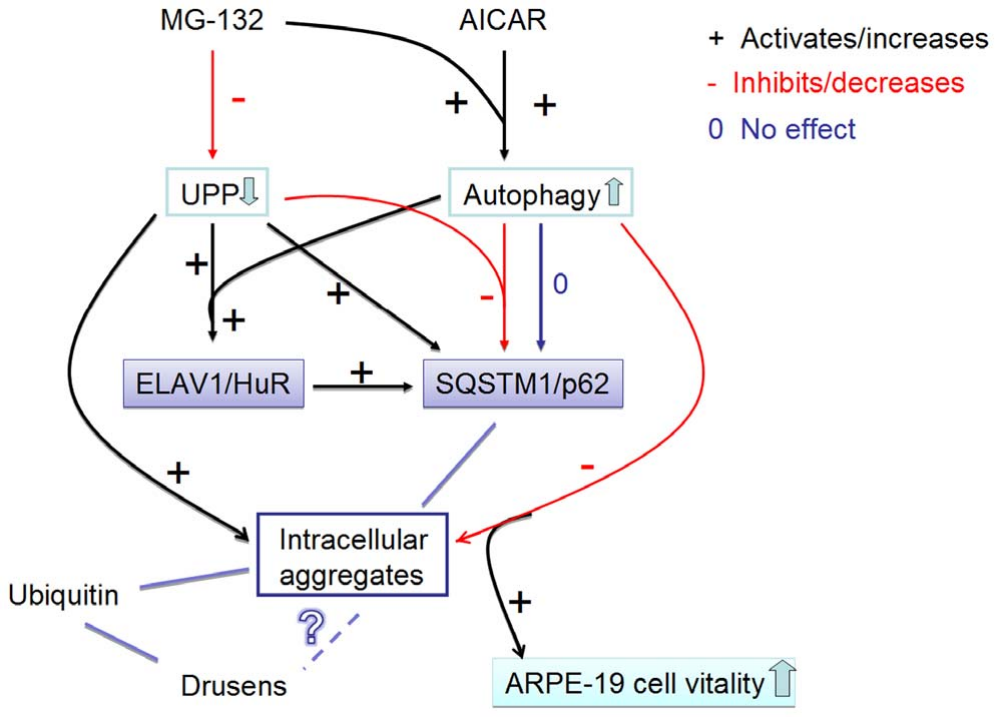
Summary of the results. The proteasome inhibitor MG-132 down-regulates (−) the ubiquitin-proteasome pathway (UPP). AMPK-activator AICAR and proteasome inhibitor MG-132 co-treatment activates (+) the autophagy. UPP inhibition significantly increases (+) SQSTM1/p62 protein levels, while autophagy induction during UPP inhibition robustly decreases (−) SQSTM1/p62 protein levels. Since SQSTM1/p62 localizes to aggregates, the decreasing of SQSTM1/p62 reveals its autophagy clearance. In addition, proteasome inhibition significantly increases ELAVL1/HuR protein levels by itself and during the autophagy induction (+). Proteasome inhibition induces a positive regulation of *SQSTM1/p62* expression that occurs also at post-transcriptional level via ELAVL1/HuR protein (+). Activated autophagy is able to completely abolish the MG-132-induced protein aggregation (−), which, in turn improves the cell vitality. Ubiquitin is also found in intracellular aggregates in ARPE-19 cells as well as from drusens. In contrast, SQSTM1/p62 is found only in the intracellular aggregates in ARPE-19 cells, but not in drusens. However, SQSTM1/p62 levels were high in macular area of RPE cells revealing impaired autophagy. What is the relation between drusen and intracellular aggregates remains still unknown. Red lines with minus symbol represent inhibiting and decreasing events in ARPE-19 cells. Black lines and plus symbol represent activating and increasing events in the ARPE-19 cell. Zero (0) and dark blue line indicate neutral effects in the ARPE-19 cell. UPP: ubiquitin-proteasome pathway.

In this study, we show for the first time that in ARPE-19 cells proteasome inhibition leads to increased *ELAVL1/HuR* expression by acting in a dual manner: on one side inducing ELAVL1/HuR transcription and, on the other side, decreasing its protein degradation; during proteasomal inhibition ELAVL1/HuR protein binds to *SQSTM1/p62* mRNA to potentiate post-transcriptionally *SQSTM1/p62* expression levels in ARPE-19 cells. SQSTM1/p62 protein is cleansed by autophagy after its stationary binding to perinuclear protein aggregates during proteasome inhibition. The cleansing can be accelerated by exposure to the AMPK activator AICAR, which counteracts the MG-132 -mediated damaging effects and improves survival in RPE. Interestingly, SQSTM1/p62 rather than ELAVL1/HuR protein accumulates strongly in the drusen rich macular area in human cadaver dry AMD samples - an evidence for impaired autophagy in the pathology of AMD. These major findings are schematically summarized in Figure 12.

Many previous studies have focused on to the transcriptional-dependent events that affect *SQSTM1/p62* expression, whereas little data is available on *SQSTM1/p62* post-transcriptional control. Our findings indicate that, in response to MG-132 stimulus, the elevation in *SQSTM1/p62* mRNA levels is due to the activation of specific intracellular molecular cascades that are not simply at the transcriptional level, but also involve the downstream fate of *SQSTM1/p62* transcript through the RNA-binding ELAVL1/HuR protein. Indeed, we first identified within *SQSTM1/p62* 3′-UTR primary sequence the presence of ARE, that are putative ELAV-binding sites. The *in vitro* experiment seems to confirm our prediction, demonstrating that the recombinant HuR protein binds specifically to the ARE-bearing RNA probe of *SQSTM1/p62*. The ELAVL1/HuR protein capability to bind to *SQSTM1/p62* mRNA was further validated *in vitro* by immunoprecipitation and real time qPCR experiments. In particular, the isolation of the transcripts co-immunoprecipitated with endogenous ELAVL1/HuR protein showed that this RNA binding protein had associated with *SQSTM1/p62* mRNA more prominently after proteasome inhibition in ARPE-19 cells. Proteasome inhibition caused ELAVL1/HuR protein accumulation, an increase in the amount of SQSTM1/p62 protein and mRNA and an increase of the formation of the ELAVL1/HuR protein- *SQSTM1/p62* mRNA complex. Therefore the binding of ELAVL1/HuR to a low affinity mRNA sequence, such as *SQSTM1/p62*, can best be explained by an alteration in the stoichiometric parameters regulating the mass action law. The increase of ELAVL1/HuR at both mRNA and protein levels in MG-132-treated cells suggests that the ELAVL1/HuR protein up-regulation may be due not only to proteasome inhibition but also to an effect on new ELAVL1/HuR protein synthesis.

In confirmation of the specific involvement of ELAVL1/HuR protein in *SQSTM1/p62* post-transcriptional expression regulation, we observed that the MG-132-induced increase of SQSTM1/p62 protein was blunted in ELAVL1/HuR silenced RPE cells. Accordingly, we found that, in ELAVL1/HuR-silenced cells, the MG-132 exposure leads to an up-regulation of *SQSTM1/p62* mRNA expression in a less extent than in MG-132-treated control cells, suggesting that physiologically the MG-132-mediated *SQSTM1/p62* increase occurs at both transcriptional and post-transcriptional level, the latter one via ELAVL1/HuR protein. These findings are particularly striking since post-transcriptional mechanisms are emerging as fundamental, precise regulators of gene expression in many stress-related conditions [44], [45]. Moreover, our results are in line with a report showing that, in human fibroblasts, MG-132 induces ELAVL1/HuR export to the cytoplasm, where it promotes the stabilization/translation of target mRNAs [46].

For the first time we show that, when co-treated with proteasome inhibitor MG-132, AICAR triggered autophagy in ARPE-19 cells, as indicated by the increase of both LC3 lipidation and autophagosome vesicles formation. As well as for AICAR, we found that also starvation alone cannot induce autophagy in ARPE-19 cells: indeed, both AICAR and starvation require the presence of MG-132 to trigger autophagic process. Notably, LC3II increase was detectable, respectively, after 0,5 h treatment with AICAR+MG-132, and 3 h treatment with starvation+MG-132, suggesting a different time course for autophagy activation by the two co-stimuli.

In ARPE-19 cells the MG-132–mediated increase of SQSTM1/p62 protein levels was robustly counteracted by the autophagy inducer AICAR. In contrast to SQSTM1/p62, the MG-132-triggered accumulation of ELAVL1/HuR protein was not affected by AICAR treatment, pointing to different proteolytic mechanisms between SQSTM1/p62 and ELAVL1/HuR: one being decreased in autophagy and the other involving the proteasomal pathway, respectively. Accordingly, a previous observation in another human cellular model reported that HuR could undergo proteasome mediated proteolysis [47].

Moreover, by using pDendra2-hLC3 (MAP1LC3A/LC3) and pDendra2-hp62 (SQSTM1/p62) plasmids, with which the position of the protein in the cell can be detected from a desired time point, we analyzed protein trafficking in cells [48], showing that both molecules involved in autophagy simultaneously disappeared via induction of autophagy in response to MG-132 and AICAR co-treatment. As far as we are aware, this is the first time that the binding of SQSTM1/p62 protein to perinuclear aggregates has been revealed as an irreversible process. We recently documented that when *SQSTM1/p62* mRNA was silenced, ubiquitin localization in perinuclear aggregates and autophagosome formation were disturbed in ARPE-19 cells; here we show that AICAR treatment alone does not decrease SQSTM1/p62 protein levels, indicating that selective SQSTM1/p62 degradation in autophagy is favoured when ubiquitinated aggregates are present, as previously documented [49], [21]. It is noteworthy, that AICAR significantly decreased protein aggregates and improved survival in MG-132-treated RPE cells, providing clear evidence for an AICAR-mediated protective effect in this stressful condition. AICAR is a well-known autophagy inducer which acts via AMPK signalling, although it may well have other effects in different experimental models [50]–[55].

Interestingly, it has been demonstrated that in human AMD donor samples both accumulations of autophagic markers and decreased lysosomal activity can be observed [56]. Furthermore, an effective autophagic clearance system has been recently documented in human RPE cells [21], [57]–[59]. During aging, lipofuscin accumulates in lysosomes – this is evidence for a declining cellular capability to degrade proteins [60]. In addition, lipofuscin promotes the misfolding of intracellular proteins, which exerts an additional oxidative stress in the RPE cells [61], [62].

As recently suggested by our group [21] and here confirmed in detail, the SQSTM1/p62 clearance via autophagy can be prevented by the lysosome inhibitor bafilomycin. Under bafilomycin treatment, an intense autophagosome formation, in parallel with SQSTM1/p62 accumulation, can be observed, further confirming SQSTM1/p62 as agood marker for impaired autophagy.

Our human AMD samples here studied show a significant elevation of SQSTM1/p62 protein levels in the macula, strongly indicating that SQSTM1/p62 accumulation may be an index of impaired autophagy. In addition, most of the drusen of AMD patients were strongly ubiquitin positive, but staining for SQSTM1/p62 was observed only intracellularly. This might imply that SQSTM1/p62 is mostly degraded by the autophagic pathway and, unlike ubiquitin, SQSTM1/p62 is not exocytosed to the extracellular space of the RPE cells [6], [63], [56]. Recently published papers have reported that ubiquitin and SQSTM1/p62 are strongly co-localized in the perinuclear aggregates [19], [21], but here we could detect only ubiquitin in the extracellular drusen of AMD samples. ELAVL1/HuR protein level staining in human dry AMD macula was rather reduced, although not significantly. Since ELAVL1/HuR is degraded in proteasomes, our findings provide evidence that proteasomes are active in these AMD cases, even though proteasomal activity may decrease during aging in the RPE cells [62], [64], [65]. As a confirmation of our hypothesis, the foveomacular areas from control no-AMD patients showed weak staining for SQSTM1/p62, ELAVL1/HuR, ubiquitin proteins, comparable to those observed in perimacular and peripheral retina of AMD patients.

Besides many recent advances in the understanding of autophagy mechanisms, it is now widely accepted that dysfunctions in these processes have a key role in many neurodegenerative diseases, including AMD. Moreover, autophagy is increasingly emerging as a novel target of therapies aimed to counteract protein aggregation and improve cell viability; within this context, preservation of autophagy may protect RPE cell from degeneration and thus represents a potential tool to slow or even prevent the development of AMD [66].

## Supporting Information

Figure S1 MG-132 induces ELAVL1/HUR and SQSTM1/p62 accumulation. A densitometric analysis of SQSTM1/p62 (A) and ELAVL1/HuR (B) proteins in the nuclear/perinuclear compartment (left panels) and in the cytoplasm (right panels) of ARPE-19 cells after exposure to MG-132 (5 µM) for 24 h. Control cells were exposed to solvent (DMSO). α-tubulin was used as a loading control. Results are expressed as means ± S.E.M.; *p<0.05, **p<0.01, ***p<0.001, control vs. treated cells, Student’s t test; n = 7.
(TIF)

Figure S2 The 24 h MG-132-mediated upregulation of *SQSTM1/p62* mRNA expression is counteracted by the ELAVL1/HuR silencing. Determination of *ELAVL1/HuR* (A) and *SQSTM1/p62* (B) mRNA levels by real-time qPCR in the total homogenates of control (CTR), MG-132-treated cells (MG-132), and MG-132-treated ELAVL1/HuR silenced ARPE-19 cells (*ELAVL1/HuR*-siRNA+MG-132). *ELAVL1/HuR* and *SQSTM1/p62* mRNA levels in control cells were taken as 100% in (A) and (B), respectively. Control cells were exposed to solvent (DMSO). The values obtained from total cellular mRNA have been normalized to the level of *RPL6* mRNA and expressed as mean ± S.E.M. ***p<0.001 control vs. treated cells; ¤¤¤p<0.001 *ELAVL1/HuR*-siRNA+MG-132 vs. MG-132; Tukey’s multiple comparison test; n = 3.
(TIF)

Figure S3 AICAR is well-tolerated and prevents cell death in MG-132 long-exposed ARPE-19 cells. (A): Maximum release of lactate dehydrogenase (LDH) enzyme in ARPE-19 cells treated with MG-132 5 µM and/or AICAR 2 mM for 24 h. Celastrol (10 µM) treatment is reported as a positive control since it induces maximum LDH release. Results are presented as mean optical density (OD) ± S.E.M. (n = 6, Mann-Whitney). (B): Cell death of ARPE-19 cells treated with MG-132 5 µM and/or AICAR 2 mM up to 72 h. Percent cell death of ARPE-19 cells (early apoptotic or annexin V-FITC+ cells −black bar- and late apoptotic or annexin V-FITC/propidium iodide+ cells −white bar) under different treatments. Data shown are mean+S.D. For late apoptosis ***p<0.001 Student’s t test; n = 3. For early apoptosis ¤¤p<0.01, ¤¤¤p<0.001 Student’s *t* test; n = 3.
(TIF)
