# Supplementary figures and images for: Autophagy Activation Clears ELAVL1/HuR-Mediated Accumulation of SQSTM1/p62 during Proteasomal Inhibition in Human Retinal Pigment Epithelial Cells
Source: PLoS One. 2013 Jul 29;8(7):e69563. doi: 10.1371/journal.pone.0069563 (PMC3726683; doi:10.1371/journal.pone.0069563)

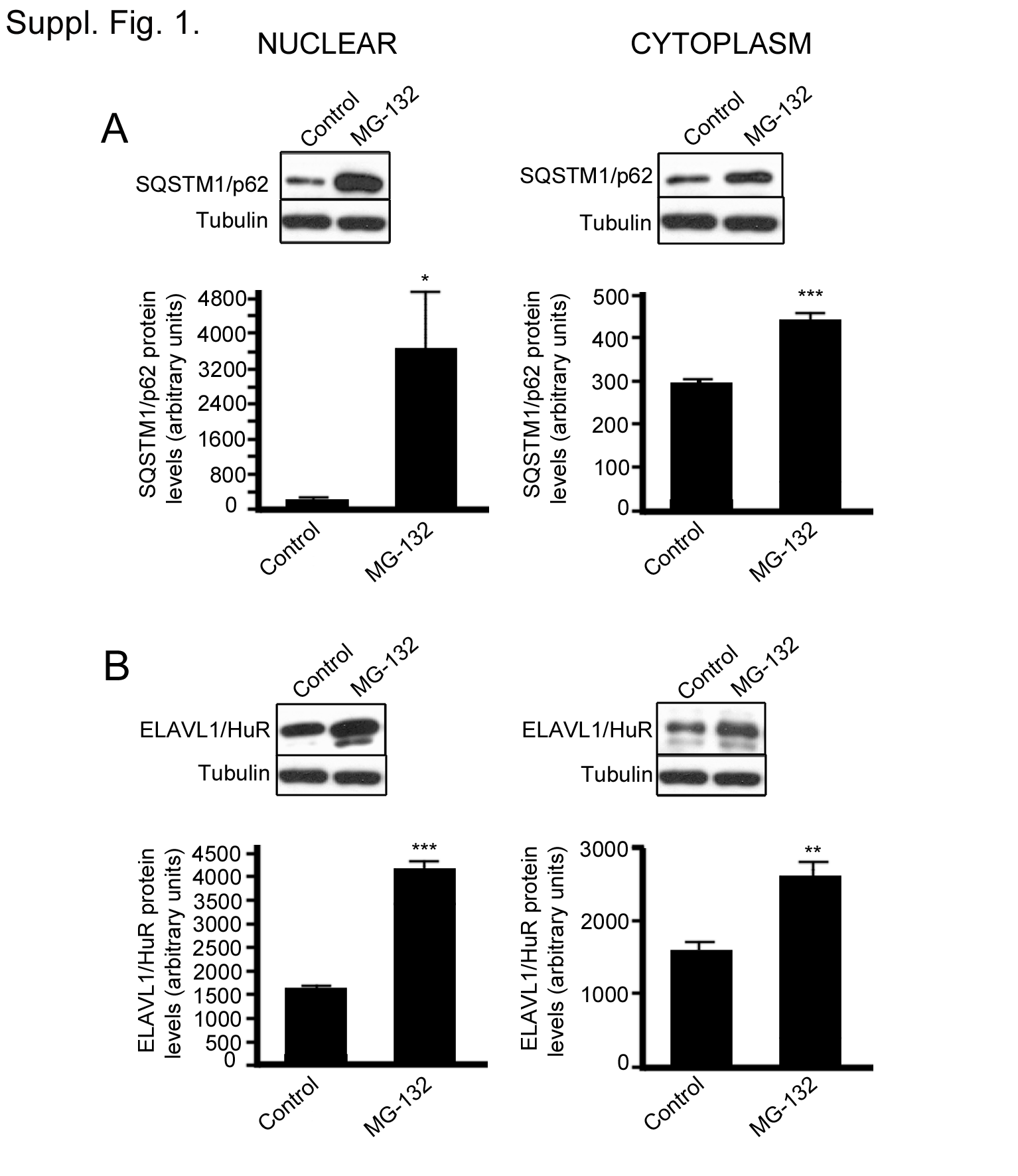

Supplement: Figure S1 — MG-132 induces ELAVL1/HUR and SQSTM1/p62 accumulation. A densitometric analysis of SQSTM1/p62 (A) and ELAVL1/HuR (B) proteins in the nuclear/perinuclear compartment (left panels) and in the cytoplasm (right panels) of ARPE-19 cells after exposure to MG-132 (5 µM) for 24 h. Control cells were exposed to solvent (DMSO). α-tubulin was used as a loading control. Results are expressed as means ± S.E.M.; *p<0.05, **p<0.01, ***p<0.001, control vs. treated cells, Student’s t test; n = 7. (TIF) [file pone.0069563.s001.tif]

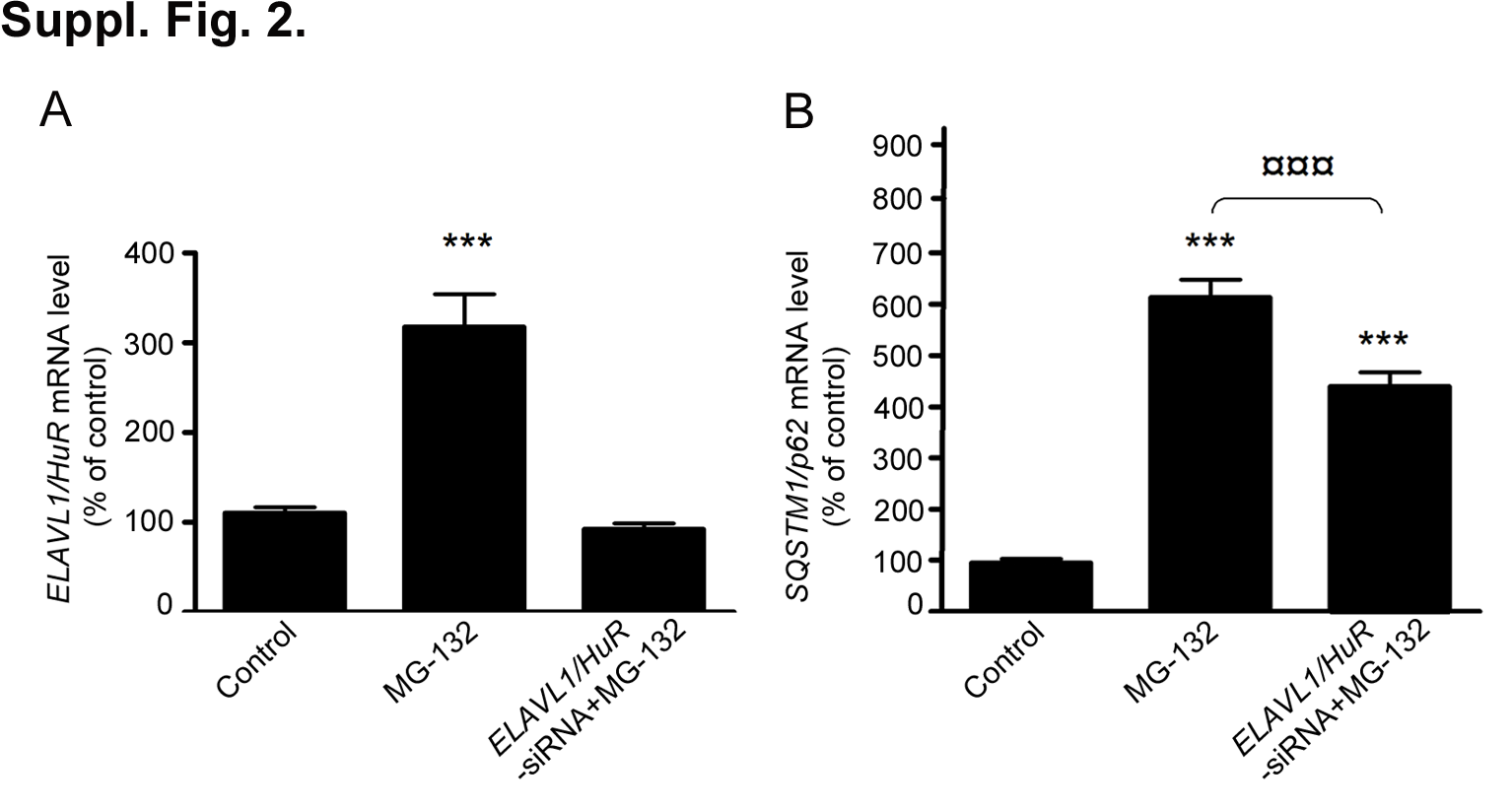

Supplement: Figure S2 — The 24 h MG-132-mediated upregulation of SQSTM1/p62 mRNA expression is counteracted by the ELAVL1/HuR silencing. Determination of ELAVL1/HuR (A) and SQSTM1/p62 (B) mRNA levels by real-time qPCR in the total homogenates of control (CTR), MG-132-treated cells (MG-132), and MG-132-treated ELAVL1/HuR silenced ARPE-19 cells (ELAVL1/HuR-siRNA+MG-132). ELAVL1/HuR and SQSTM1/p62 mRNA levels in control cells were taken as 100% in (A) and (B), respectively. Control cells were exposed to solvent (DMSO). The values obtained from total cellular mRNA have been normalized to the level of RPL6 mRNA and expressed as mean ± S.E.M. ***p<0.001 control vs. treated cells; ¤¤¤p<0.001 ELAVL1/HuR-siRNA+MG-132 vs. MG-132; Tukey’s multiple comparison test; n = 3. (TIF) [file pone.0069563.s002.tif]

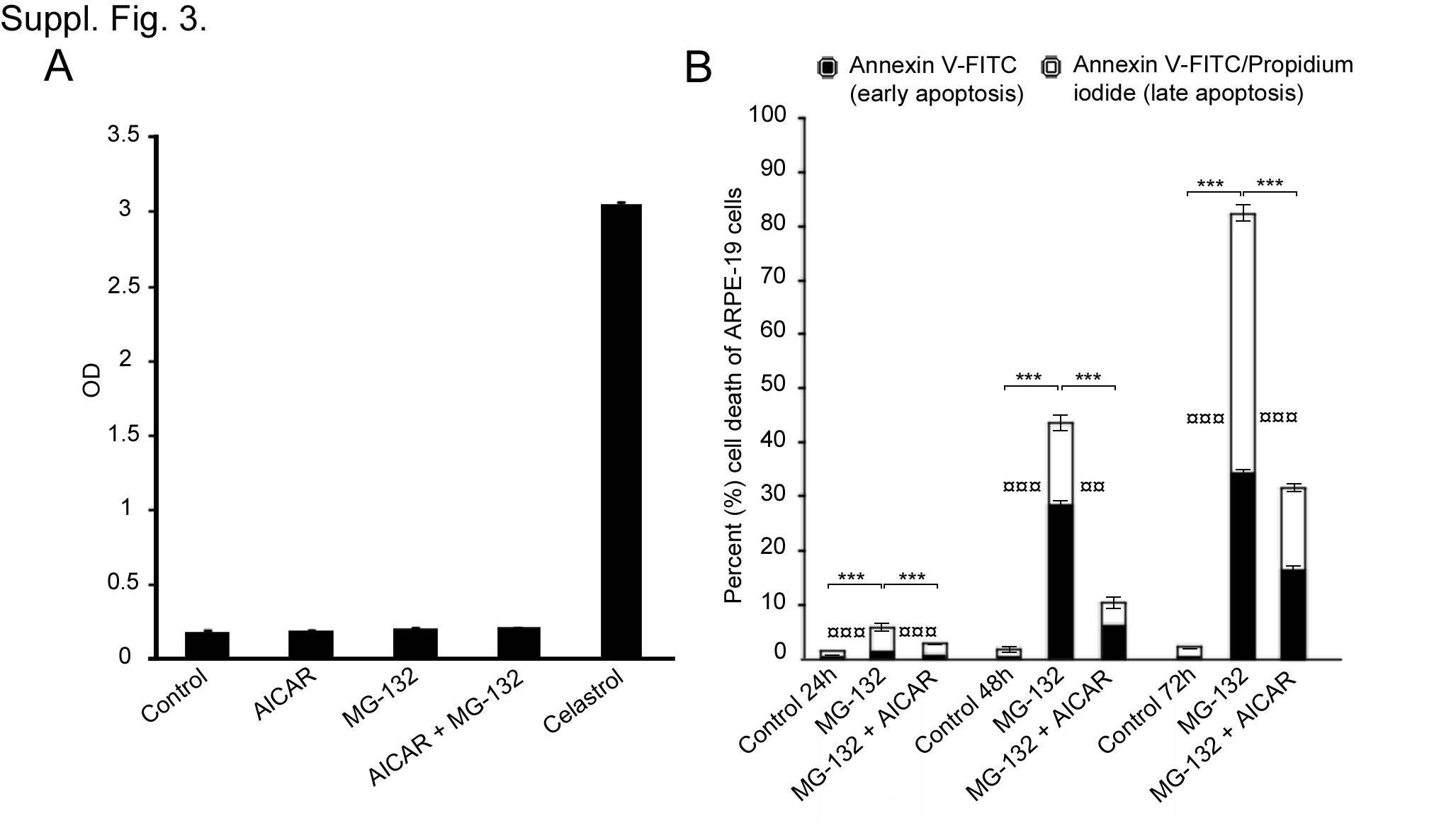

Supplement: Figure S3 — AICAR is well-tolerated and prevents cell death in MG-132 long-exposed ARPE-19 cells. (A): Maximum release of lactate dehydrogenase (LDH) enzyme in ARPE-19 cells treated with MG-132 5 µM and/or AICAR 2 mM for 24 h. Celastrol (10 µM) treatment is reported as a positive control since it induces maximum LDH release. Results are presented as mean optical density (OD) ± S.E.M. (n = 6, Mann-Whitney). (B): Cell death of ARPE-19 cells treated with MG-132 5 µM and/or AICAR 2 mM up to 72 h. Percent cell death of ARPE-19 cells (early apoptotic or annexin V-FITC+ cells −black bar- and late apoptotic or annexin V-FITC/propidium iodide+ cells −white bar) under different treatments. Data shown are mean+S.D. For late apoptosis ***p<0.001 Student’s t test; n = 3. For early apoptosis ¤¤p<0.01, ¤¤¤p<0.001 Student’s t test; n = 3. (TIF) [file pone.0069563.s003.tif]
